# Mesothelin blockage by Amatuximab suppresses cell invasiveness, enhances gemcitabine sensitivity and regulates cancer cell stemness in mesothelin-positive pancreatic cancer cells

**DOI:** 10.1186/s12885-020-07722-3

**Published:** 2021-02-26

**Authors:** Fumihiko Matsuzawa, Hirofumi Kamachi, Tatsuzo Mizukami, Takahiro Einama, Futoshi Kawamata, Yuki Fujii, Moto Fukai, Nozomi Kobayashi, Yutaka Hatanaka, Akinobu Taketomi

**Affiliations:** 1 Department of Gastroenterological Surgery I, Hokkaido University Graduate School of Medicine, North 15, West 7, Kita-Ku, Sapporo, Hokkaido 060-8638 Japan; 2 Department of Surgery, National Defense Medical College, Namiki 3-2, Tokorozawa, Saitama, 359-8513 Japan; 3 Research Division of Companion Diagnostics, Hokkaido University Hospital, Kita 14, Nishi 5, Kita-ku, Sapporo, Hokkaido 060-8638 Japan

**Keywords:** Amatuximab, Mesothelin, Peritoneal metastasis, Cancer stem cell, Pancreatic cancer, pMET, C-MET

## Abstract

**Background:**

Mesothelin is a 40-kDa glycoprotein that is highly overexpressed in various types of cancers, however molecular mechanism of mesothelin has not been well-known. Amatuximab is a chimeric monoclonal IgG1/k antibody targeting mesothelin. We recently demonstrated that the combine therapy of Amatuximab and gemcitabine was effective for peritonitis of pancreatic cancer in mouse model.

**Methods:**

We discover the role and potential mechanism of mesothelin blockage by Amatuximab in human pancreatic cells both expressing high or low level of mesothelin in vitro experiment and peritonitis mouse model of pancreatic cancer.

**Results:**

Mesothelin blockage by Amatuximab lead to suppression of invasiveness and migration capacity in AsPC-1 and Capan-2 (high mesothelin expression) and reduce levels of pMET expression. The combination of Amatuximab and gemcitabine suppressed proliferation of AsPC-1 and Capan-2 more strongly than gemcitabine alone. These phenomena were not observed in Panc-1 and MIA Paca-2 (Mesothelin low expression). We previously demonstrated that Amatuximab reduced the peritoneal mass in mouse AsPC-1 peritonitis model and induced sherbet-like cancer cell aggregates, which were vanished by gemcitabine. In this study, we showed that the cancer stem cell related molecule such as ALDH1, CD44, c-MET, as well as proliferation related molecules, were suppressed in sherbet-like aggregates, but once sherbet-like aggregates attached to peritoneum, they expressed these molecules strongly without the morphological changes.

**Conclusions:**

Our work suggested that Amatuximab inhibits the adhesion of cancer cells to peritoneum and suppresses the stemness and viability of those, that lead to enhance the sensitivity for gemcitabine.

**Supplementary Information:**

The online version contains supplementary material available at 10.1186/s12885-020-07722-3.

## Background

Pancreatic cancer shows rapid growth and metastasis and is one of the most fatal human cancers. Over half of patients are diagnosed at a stage where metastases have developed, and the overall 5-year survival rate for the pancreatic patients with metastases is only 10% [1, 2]. Only 15–20% of patients have resectable disease at the time of diagnosis [3]. The majority of patients have local and distant micrometastases at the time of surgery. Therefore, disease recurrence following operation is very high. Hattangadi et al. reported that the peritoneum (55%) and the liver (53%) were the most common sites of recurrence [4].

Adjuvant therapies such as radiotherapy and chemotherapy (or a combination) help improve the prognosis of pancreatic cancer. However, their effectiveness is limited. Gemcitabine [5] or S-1 [6] is the most common adjuvant chemotherapy for pancreatic cancer after surgery. Recent studies showed that in patients with advanced disease, combining gemcitabine with other systemic agents can improve patient outcome compared with standard gemcitabine monotherapy [7]. For example, the combined therapy with gemcitabine plus albumin-bound paclitaxel reached 8.7 months of median overall survival in phase III trial [2]. Although the prognosis of this disease has been improved, these results were not acceptable for the patients in clinical. Thus, the identification of novel and effective strategies is critical to improve the prognosis for pancreatic cancer patients.

Mesothelin is a 40-kDa glycosylphosphatidyl inositol-linked protein that is expressed in mesothelial cells lining the peritoneum among the human normal tissues [8]. Mesothelin is highly overexpressed in various types of cancers, such as mesothelioma [8], ovarian [8] and pancreatic cancer [9]. Recent studies have demonstrated a role for mesothelin in cell survival, cell migration, cell invasion and tumor progression [10]. The expression of mesothelin is also related to epithelial-mesenchymal transition (EMT) and cancer stem cells (CSCs) [11]. We previously reported the significance of mesothelin expression in the poor prognosis of gastrointestinal cancer patients [12–17]. Because of the expression pattern, mesothelin has recently been targeted for immunotherapy [18].

Amatuximab is an investigational chimeric high-affinity monoclonal IgG1/k antibody that targets mesothelin [19]. In vitro, Amatuximab elicits antibody-dependent cellular cytotoxicity against mesothelin-expressing tumor cell lines and inhibits heterotypic cell adhesion of mesothelin-positive tumor cells to CA125-expressing tumor cells. Based on its safety in a Phase I study and preclinical studies showing synergy with chemotherapy [20], Amatuximab was combined with gemcitabine in a Phase II study (NCT00570713) in patients with previously untreated unresectable stage 3 or 4 pancreatic cancer. However, the study did not demonstrate the efficacy of combined therapy compared with either Amatuximab or chemotherapy alone. The trial included patients with unresectable bulky pancreatic cancer, and we suspect that Amatuximab could not be delivered to pancreatic tumor cells in these patients.

We previously reported that Amatuximab monotherapy reduced the peritoneal mass volume in peritonitis model mouse of human pancreatic cancer cells and observed the appearance of sherbet-like aggregates in this model [21]. We demonstrated that the combined therapy using Amatuximab and gemcitabine reduced the peritoneal mass and eliminated the sherbet-like aggregates. However, the molecular mechanism of this phenomenon is not yet understood.

The aim of this study is to clarify the role and potential mechanism of mesothelin receptor blockage by Amatuximab in human pancreatic cell lines both in vitro and in vivo*.*

## Methods

### Cell culture

The human pancreatic adenocarcinoma AsPC-1 and Capan-2 cell lines were cultured in RPMI-1640 medium supplemented with 10% FBS, 100 units/ml penicillin and 100 μg/ml streptomycin. Panc-1 and MIA Paca-2 cell lines were cultured in DMEM medium supplemented with 10% FBS, 100 units/ml penicillin and 100 μg/ml streptomycin. The cells were cultured and maintained as previously described [21]. We obtained AsPC-1 cells (ATCC® CRL-1682™) in 2010, MIA Paca-2 (ATCC® CRL-1420™) cells and Panc-1 (ATCC® CRL-1469™) cells in 2016 and Capan-2 cells (ATCC® HTB-80™) in 2019. All cell lines were obtained from ATCC (Manassas, VA, USA). After obtaining cells from ATCC, cells were frozen at low passage (p3-p5), and then thawed and used at passage 10 or lower. These cell lines were tested for mycoplasma contamination at regular intervals. we did not authenticate these cell lines.

### Reagents

Amatuximab (also known as MORAb-009) was obtained from MORPHOTEK INC (Exton, PA, USA). Gemcitabine hydrochloride (Gemzar) was purchased from Eli Lilly (Indianapolis, IN, USA).

### Proliferation assay (cell counting method)

Pancreatic cancer cell lines were seeded at a density of 1.5 × 10^5^ cells per 2 mL medium in a 35 mm plate, and Amatuximab was added at various concentrations (0, 0.01, 1 or 100 μg/mL). After 48 h, cells were harvested by Trypsin/EDTA (0.05%) (Thermo Fisher Scientific Inc., Waltham, MA, USA). Next, 50 μL of the cell suspension were mixed with 50 μL of 0.4% trypan blue (#207–17,081, Wako, Tokyo, Japan) by gentle pipetting and then 20 μL of the mix were loaded into a chamber in a hemocytometer. Counts were performed in triplicate by one analyst under a 40× objective according to standard methodology using BIOREVO (#BZ-9000, KEYENCE, Osaka, Japan).

### Invasion assay

In vitro invasion assays were performed using the BD Bio-Coat Matrigel invasion assay system (BD Biosciences, San Jose, CA, USA) according to the manufacturer’s instructions. Briefly, cells were seeded into Matrigel-precoated Transwell chambers consisting of polycarbonate membranes with 8.0 μm pores. The Transwell chambers were then placed into 24-well plates, into which basal medium only or basal medium containing 100 μg/mL Amatuximab or control IgG was added. We decided the concentration of Amatuximab or control IgG from our data in proliferation assay and the past literature, which showed the strong blockage of adhesion between mesothelin and CA125 in this concentration of Amatuximab [19]. After incubating cells for 24 h, the upper surface of the Transwell chambers was wiped with a cotton swab and the invading cells were fixed and stained using the Diff-Quick cell staining kit (Dade Behring, Inc., Newark, DE, USA). We counted the number of invading cells in five random microscopic fields (200× magnification) using BIOREVO (#BZ-9000, KEYENCE, Osaka, Japan).

### Migration assay

In vitro migration assays were performed using the BD Bio-Coat migration assay system (BD Biosciences) according to the manufacturer’s instructions and the methodology for the invasion assays.

### Cell growth inhibition assay

Pancreatic cancer cells were seeded at a density of 1.5 × 10^5^ cells per 35 mm dish in culture medium containing 10% FBS with 1 μM gemcitabine and 100 μg/ml Amatuximab or control IgG. After 48 h, the numbers of viable cells were counted as described in the previous section. The concentration of gemcitabine was determined based on previous studies [22] and our preliminary experiments. Counts were performed in triplicate by one analyst under a 40× objective according to standard methodology using BIOREVO (#BZ-9000, KEYENCE, Osaka, Japan).

### Western blot analysis

Cells were seeded at a density of 1.5 × 10^6^ cells in 2 ml medium in a 100 mm dish. Amatuximab (100 μg/ml) or control IgG was added to medium at the time of seeding and cells were cultured for 24 or 48 h. After cells were harvested, western blot analysis was performed as previously described [21]. The cells were homogenized at 4 °C in lysis buffer (0.1% SDS, 1% Igepal CA-630, 0.5% sodium deoxycholate) and a protease inhibitor cocktail (Sigma-Aldrich, St. Louis, MO, USA). Cell lysates (20–50 μg) were resolved by electrophoresis on polyacrylamide gels and transferred to PVDF membranes (Millipore, Billerica, MA, USA). After blocking the membranes in 5% non-fat dry milk or 3% bovine serum albumin in Tris-buffered saline for 1 h at room temp, the blots were hybridized overnight at 4 °C with primary antibodies. After hybridization with secondary antibodies conjugated with HRP (Cell Signaling Technology, Danvers, MA, USA), immunocomplexes were visualized using an enhanced chemiluminescence kit (GE Healthcare, Chalfont St. Giles, UK). Densitometric analysis of western blots was performed using a ChemiDoc XRS Plus system with Image Lab Software (Bio-Rad, Hercules, CA, USA). Primary antibodies are described in Supplemental Table 1.

### FACS analysis

We harvested, washed and resuspended the cells in ice-cold PBS. Primary antibody was added at 2 μg/ml and incubated for 1 h on ice. Cells were then washed with PBS, incubated with Alexa Fluor 488 goat-anti-mouse secondary antibody (Invitrogen, Carlsbad, CA, USA) diluted to 1 μg/ml, washed as above and analyzed on BD FACS Canto™ II flow cytometer (BD Biosciences).

### Animals

Six-week-old female BALB/c Slc-nu/nu mice (body weight 14-19 g) were purchased from CLEA Japan, INC. (Tokyo, Japan). Mice were maintained under specific pathogen-free conditions in laminar-flow benches and were allowed to adapt to the environment for 1 or 2 weeks before experiments. All procedures involving animals and their care were approved by the Institutional Animal Care and Use Committee of National University Corporation Hokkaido University and were conducted under National University Corporation Hokkaido University Regulations on Animal Experimentation.

### Generation of peritoneal model mouse using AsPC-1 cells

We generated the peritoneal model mouse as described previously [21]. Briefly, 5 × 10^6^ AsPC-1 cells were injected to the abdominal cavity of 7-week-old nude mice. The mice were divided to two groups randomly. Amatuximab (200 mg/kg) (*n* = 6) or isotype control IgG (200 mg/kg) (*n* = 4), so totally using 10 mice, was administered by injection into the abdominal cavity every 2 days for 22 days. Two groups were maintained in the same type of cage and the environment. The mice were euthanized by isoflurane inhalation followed by cervical dislocation on day 22 and peritoneal masses and sherbet-like aggregations were harvested. No expected or unexpected adverse events happened. The resected specimens were fixed with formalin for 6 h immediately after harvest and embedded in paraffin, and then sliced sequentially at a thickness of 3 μm. We established the humane endpoints that when the model mouse got poor physical condition during the experiment, we planned to euthanize by isoflurane inhalation followed by cervical dislocation. No animals were adapted to the criteria. We set no specific criteria used for including and excluding animals during the analysis. The first author understood the group allocation totally through the experiment. These protocols were prepared before the study.

### Immunohistochemistry

We performed the immunohistochemistry using the EnVision+ System-HRP (Dako Japan, Tokyo), in the same method as described previously [21]. Briefly, the sections were mounted on glass slides, deparaffinized, and rehydrated through several graded ethanol. We retrieved the antigens in Dako EnVision FLEX Target Retrieval Solution low pH using Dako PT Link for 20 min at 97 °C according to the manufacturer’s instructions (Dako, Japan). After we blocked the endogenous peroxidase activity with 0.03% hydrogen peroxide, the tissue sections were incubated with primary antibodies at room temp for 30 min and then we reacted the samples with a dextran polymer reagent combined with secondary antibodies and peroxidase for 30 min at room temperature. Specific antigen-antibody reactions were visualized with diaminobenzidine chromogen applied for 10 min. After that, these slides were stained with hematoxylin, dehydrated and mounted. The primary antibodies are described in Supplemental Table 1. The percentage of stain-positive cells and strength of staining were evaluated in the analysis. The representative images were taken using OLYMPUS equipment described below (microscope BX63–44-FLD-2, Camera DP80, software cellSens, OLYMPUS, Tokyo, Japan).

### Quantitative RT-PCR

Total RNA from tissues and cells was isolated using TRIzol reagent (Invitrogen). Relative levels of E-cadherin, ALDH1, CD44 and c-MET mRNA were examined using SYBR Green Real-time Quantitative RT-PCR (qRT-PCR) (LightCycler 480 Roche, Switzerland) and were normalized to levels of GAPDH mRNA. We described the primers in Supplemental Table 2. Relative expression was calculated using the 2^-ΔΔCT^ method. All qRT-PCR analyses were carried out in triplicate, and the data are presented as means±standard of the means.

### Statistical analysis

Data are shown as mean ± SD. Significance of differences was determined by Student’s t-test, one-way ANOVA, two-way ANOVA or Mann-Whitney U-test. *P* < 0.05 was considered statistically significant. All statistical procedures were performed using JMP Pro 10 (SAS Institute, Japan).

## Results

### Comparison of mesothelin expression in four pancreatic cancer cell lines

**Fig. 1:**
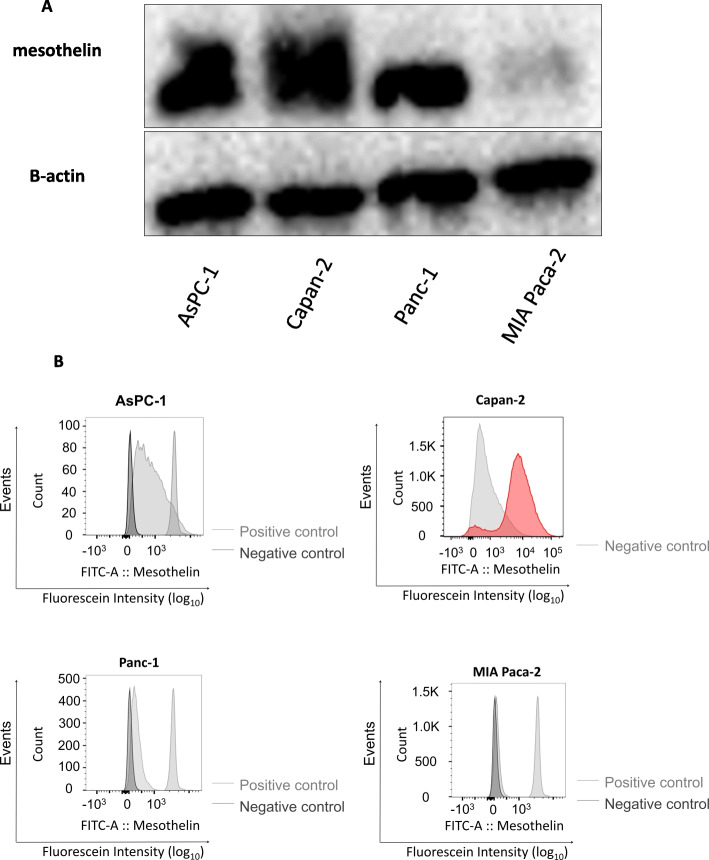
Comparison of mesothelin expression in human pancreatic cancer cell lines. **a** Whole cell lysates of AsPC-1, Capan-2, Panc-1 and MIA Paca-2 cells were analyzed for mesothelin expression by western blotting. β-actin was used as an internal control. Full-length blots/gels are presented in Supplemental Figure 2. Densitometric analysis of western blots was performed using a ChemiDoc XRS Plus system with Image Lab Software (Bio-Rad, Hercules, CA, USA). **b** FACS analysis of mesothelin expression on the surface of the four human pancreatic cancer cell lines. Onecomp eBeads (#01–1111, eBioscience) is used for positive control and FITC Mouse IgG1κ Isotype Control (#555748, BD Biosciences) is used for negative control. The results of analysis of mesothelin expression in the four human pancreatic cancer cells by immunocytochemistry were shown in Supplementary Figure 2

We first evaluated mesothelin expression in AsPC-1, Capan-2, Panc-1 and MIA Paca-2 pancreatic cell lines. Western blotting analysis revealed that Capan-2 cells showed the strongest expression of mesothelin among the four cell lines. Although the expression of mesothelin in AsPC-1 cells was weaker than that in Capan-2, AsPC-1 showed the stronger expression than those in Panc-1 and MIA Paca-2 cells. Although the expression of mesothelin in Panc-1 was detected, that was weak compared with those in Capan-2 and AsPC-1 (Fig. 1a). The result using another primary antibody for mesothelin showed that the mesothelin expression in Panc-1 was low level (Supplemental Figure 1), and the order of expression level was same in both results. FACS (Fig. 1b) and immunocytochemistry analysis (Supplemental Figure 7) of mesothelin expression in the four pancreatic cell lines were consistent with the western blot analysis. In consideration of these results totally, we thus selected AsPC-1 and Capan-2 cells as pancreatic cancer cell lines strongly expressing mesothelin (mesothelin-high), and Panc-1 and MIA Paca-2 cells as cell lines expressing low levels of mesothelin (mesothelin-low) for subsequent in vitro experiments.

### Suppression of invasiveness and migration capacity in mesothelin-high pancreatic cancer cell lines by Amatuximab treatment

**Fig. 2:**
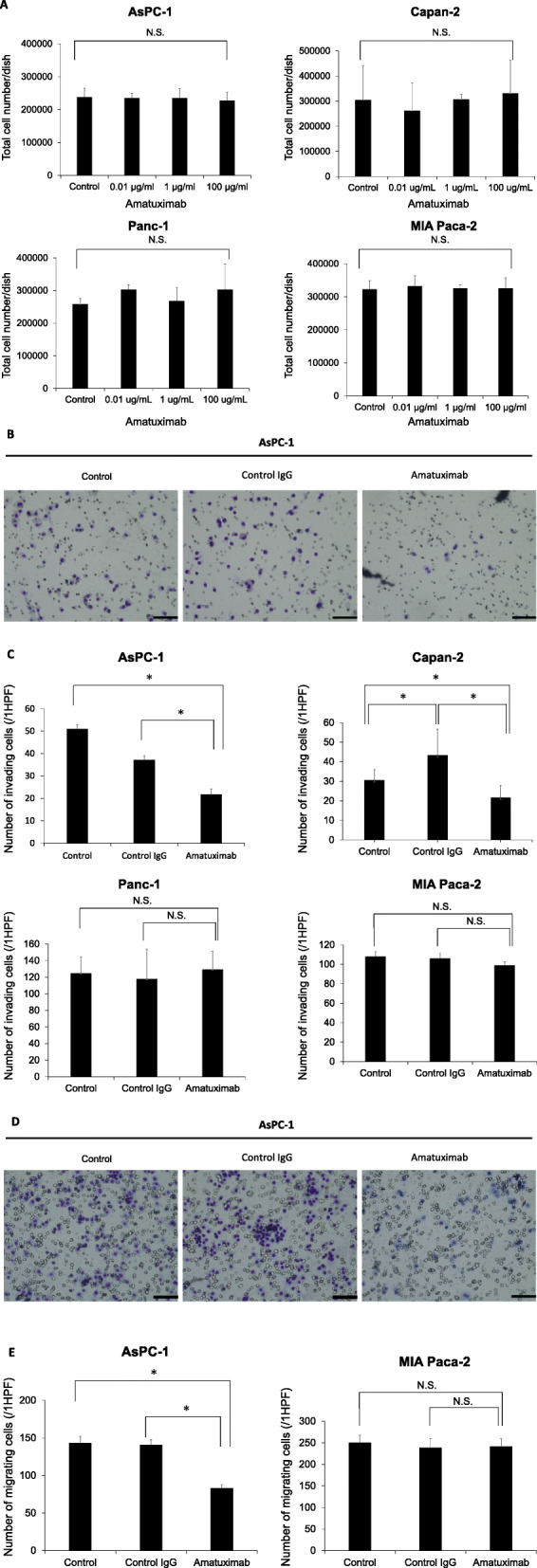
Effect of Amatuximab on pancreatic cancer cell proliferation, invasion and migration. **a** AsPC-1, Capan-2, Panc-1 and MIA Paca-2 cells were incubated with the indicated concentrations of Amatuximab for 48 h. Viable cells were stained with trypan blue and counted. N.S., not significant. **b**, **c** Invasion assays were performed in AsPC-1, Capan-2, Panc-1 and MIA Paca-2 cells treated with Amatuximab (100 μg/mL) or control IgG (100 μg/mL) for 13 h. **d**, **e** Migration assays were performed in AsPC-1 cells and MIA Paca-2 cells treated with Amatuximab (100 μg/mL) or control IgG (100 μg/mL) for 13 h. *P < 0.05. 1HPF: one high power field. Scale bar, 100 μm

We next examined the effects of blockade of mesothelin by Amatuximab on the proliferation of pancreatic cancer cells. AsPC-1, Capan-2, Panc-1 and MIA Paca-2 cells were treated with various concentrations of Amatuximab (0, 0.01, 1 or 100 μg/mL). Amatuximab treatment had no effect on the proliferation of either of the cell lines (Fig. 2a). We examined the effects of Amatuximab on the invasion capacities of AsPC-1, Capan-2, Panc-1 and MIA Paca-2 cells. We showed the representative images of invasion assay upon AsPC-1 cells in Fig. 2b. The results showed that the invasiveness was suppressed in AsPC-1 and Capanc-2 cells upon treatment with Amatuximab compared with control treatments. In contrast, Amatuximab had no impact on the invasion of Panc-1 and MIA Paca-2 cells (Fig. 2c). We also examined the effects of Amatuximab on the migration capacities of AsPC-1 and MIA Paca-2 cells. The representative images were shown in Fig. 2d. The results showed that the migration capacities were suppressed in AsPC-1 cells upon treatment with Amatuximab compared with control treatments. In contrast, Amatuximab had no impact on the migration capacity of MIA Paca-2 cells (Fig. 2e).

### Reduced levels of pMET expression in mesothelin-high pancreatic cell lines treated with Amatuximab

**Fig. 3:**
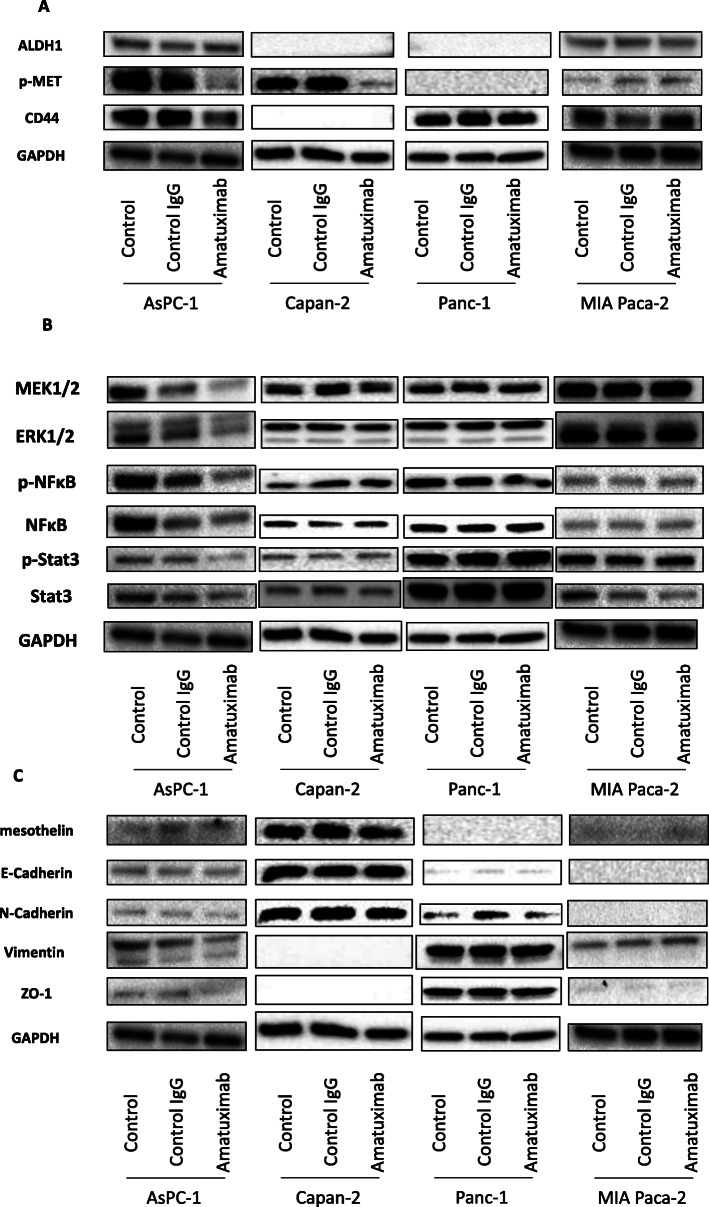
Molecular changes in human pancreatic cancer cells exposed to Amatuximab. Western blot analyses in AsPC-1, Capan-2, Panc-1 and MIA Paca-2 cells treated with control IgG or Amatuximab for the indicated proteins related to **a** cancer stem cells, **b** survival and chemoresistance, and **c** epithelial-mesenchymal transition. GADPH was used as an internal control. Ful-length blots are presented in Supplemental Figures 3, 4 and 5. Densitometric analysis of western blots was performed using a ChemiDoc XRS Plus system with Image Lab Software (Bio-Rad, Hercules, CA, USA)

We next investigated the mechanism underlying the effects of Amatuximab in pancreatic cancer cells by examining changes in molecular factors in response to Amatuximab treatment using western blotting analysis. We examined a panel of CSC-related molecules and found that the levels of p-MET were reduced in both AsPC-1 and Capan-2 cells (mesothelin-high) treated with Amatuximab compared with controls. In contrast, no changes in p-MET levels were observed in Panc-1 and MIA Paca-2 cells (mesothelin-low) treated with Amatuximab. The expression level of CD44 in AsPC-1 cells treated with Amatuximab was decreased compared with controls, however this phenomenon was not observed in Capan-2 cells and other mesothelin-low cells (Fig. 3a).

### No significant changes in expression of ERK/MEK pathway nor NFκB/Stat3 pathway proteins in mesothelin-high pancreatic cancer cells treated with Amatuximab

We also examined molecular factors related to proliferation and chemoresistance in pancreatic cell lines treated with Amatuximab. We observed the slight decrease in the expressions of MEK1/2 and ERK1/2 in AsPC-1 cells upon treatment with Amatuximab, but not in significant. Using the same approach, we examined molecules related to cell survival, invasion and migration in pancreatic cell lines treated with Amatuximab. We observed the slight decrease in the expressions of p-NFκB and p-Stat3 in AsPC-1 cells upon treatment with Amatuximab, but not in significant (Fig. 3b).

We did not detect any changes in EMT-related proteins or mesothelin expression in either cells upon Amatuximab treatment (Fig. 3c).

### Suppression on cell growth of combined Amatuximab and gemcitabine treatment in mesothelin-high pancreatic cancer cell lines

**Fig. 4:**
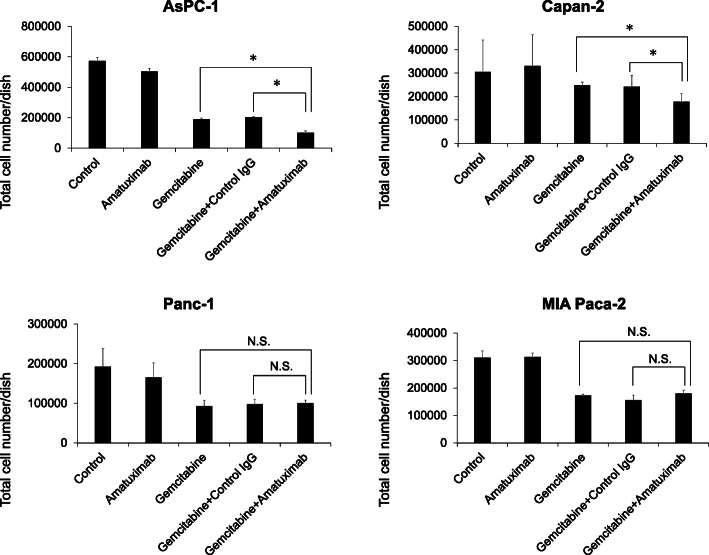
Effect of Amatuximab and gemcitabine combination treatment in human pancreatic cancer cell lines. AsPC-1, Capan-2, Panc-1 and MIA Paca-2 cells were treated with gemcitabine (1 μM) and/or Amatuximab (100 μg/mL) for 48 h. Viable cells were stained with trypan blue and counted

We next examined the effect of combination treatment with Amatuximab and gemcitabine in pancreatic cancer cell lines. The results showed that the combined therapy suppressed the proliferation of AsPC-1 and Capan-2 cells (mesothelin-high) more strongly than gemcitabine alone. Notably, these results were not observed in Panc-1 and MIA Paca-2 cells (Fig. 4).

### Suppression of E-cadherin in sherbet-like aggregates

**Fig. 5:**
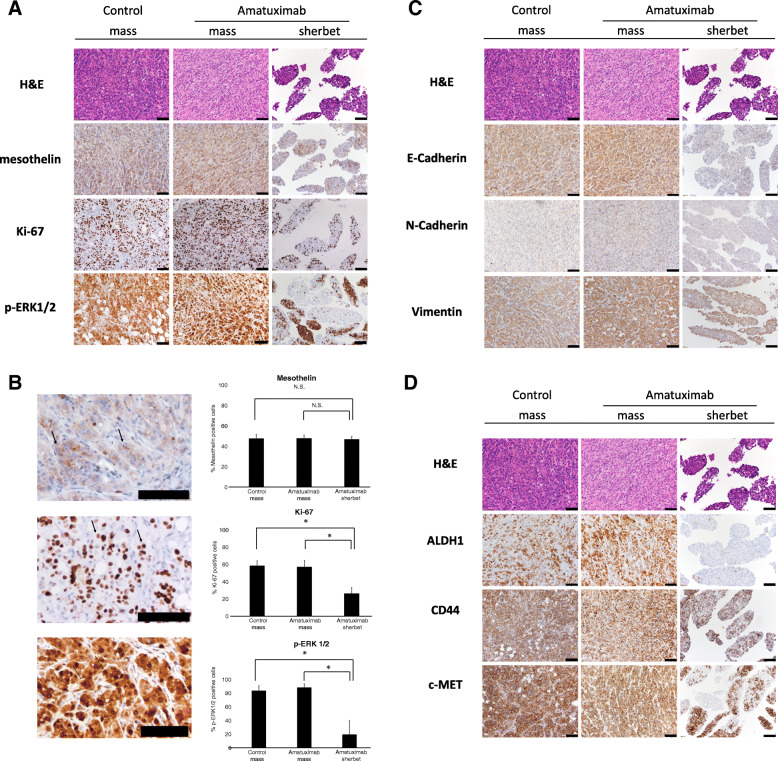

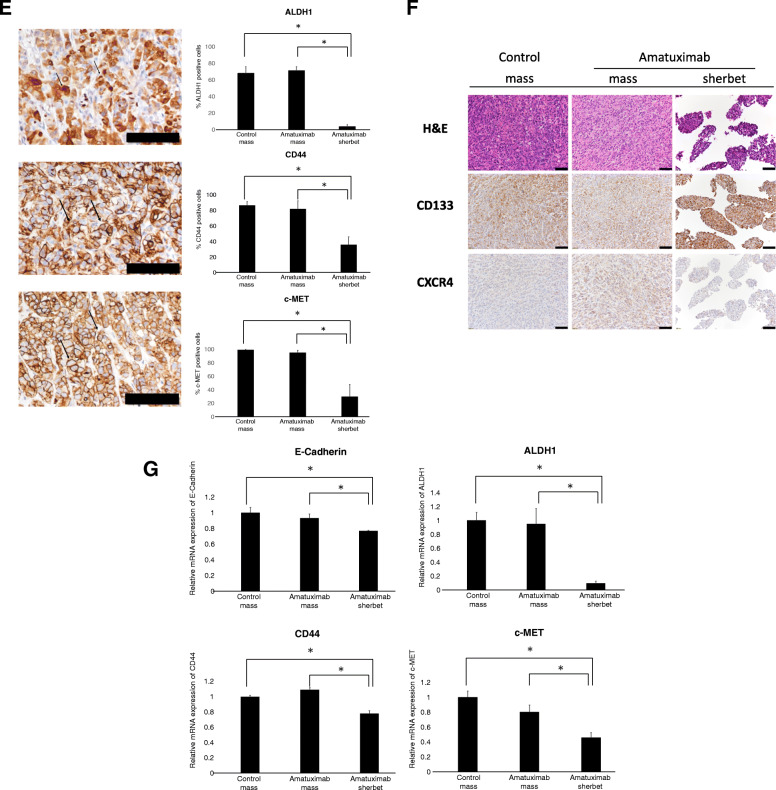
Immunohistochemical analysis in the pancreatic peritoneal dissemination model treated by Amatuximab. Immunohistochemistry was performed on indicated sections from Amatuximab or control treated model mice for proteins related with **a** chemoresistance, **c** epithelial mesenchymal transition, adhesion, **d**, **f** stemness and metastasis. **b**, **e** Quantification of positively stained cells for the indicated proteins. Arrows indicate the representative image of the positively stained cells. The frequencies of positively stained cells were counted in six high power fields that were chosen at random. **g** Quantitative PCR of the indicated factors in RNA samples harvested from blocks. *P < 0.05. Scale bar, 100 μm

We generated the mouse peritoneal dissemination model of pancreatic cancer using the previously reported protocol [21] and treated mice either with Amatuximab or isotype control IgG as described in methods. No animals were excluded during the experiment and the analysis. We examined mass lesions and sherbet like aggregates by immunohistochemistry. No changes were observed in mesothelin expression between control and Amatuximab mice (Fig. 5a). The expression of p-ERK1/2 was heterogenous in sherbet-like aggregates of Amatuximab-treated group in contrast to strong homogenous expressions in mass lesions of both Amatuximab-treated group and control group. Ki-67 expression was reduced in sherbet-like aggregates of Amatuximab-treated group compared with mass lesions of both Amatuximab-treated group and control group (Fig. 5a, b). Evaluation of EMT-related protein expression revealed no changes except for the suppression of E-Cadherin expression in Amatuximab groups (Fig. 5c).

### Suppression of CSC-related molecules in sherbet-like aggregates

We next examined the expressions of CSC-related molecules. We observed the suppression of ALDH1/c-MET/CD44 expression in sherbet-like aggregates in Amatuximab mice compared with those of mass lesions in both Amatuximab-treated group and control group (Fig. 5d, e). Expression of CXCR4 showed no change, while CD133 expression was enhanced in sherbet-like aggregates compared with those of mass lesions in both Amatuximab-treated group and control group (Fig. 5f). We also examined the mRNA expressions of these molecules, and the results were consistent with the immunohistochemistry results (Fig. 5g). These results showed that the stemness of sherbet-like aggregates in the Amatuximab group was suppressed compared with that of peritoneal metastasis tissues.

### Cancer cell clusters adhesive to the surface of peritoneal metastasis are morphologically similar to sherbet-like aggregates

**Fig. 6:**
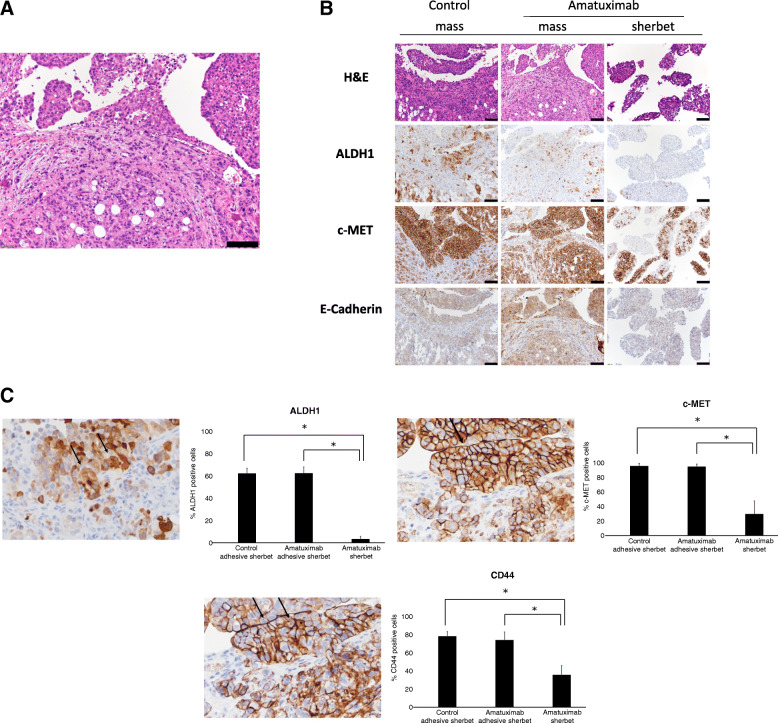
Molecular transition of sherbet like aggregates attaching to mesothelium. **a** H&E image of peritoneal mass from Amatuximab treated mice. **b** Immunohistochemistry of the indicated proteins. **c** Immunohistochemistry of the indicated proteins shown on the left; the frequencies of positive stained cells in each treatment group were calculated and shown on the right. Arrows indicate the representative image of the positively stained cells *P < 0.05. Scale bar, 100 μm

We found the cancer cell clusters adhesive to the surface of peritoneal metastasis, those were morphologically similar to the sherbet-like aggregates (Fig. 6a). The frequencies of these clusters were not related to the treatment or absence of Amatuximab (data not shown).

### Protein expressions in cancer cell clusters attached to the peritoneal metastasis tissue

We next examined the protein expressions in the cancer cell clusters by immunohistochemistry. The expressions of ALDH1, c-MET and CD44 were enhanced in cancer cell clusters attached to the metastases compared with those in sherbet-like aggregates in Amatuximab mice (Fig. 6b). The protein expressions in cancer cells adhered to metastases were more similar to those of peritoneal metastasis tissue than those of the sherbet-like aggregates (Fig. 6b, c).

### Amatuximab inhibits the adhesion of cancer cells to peritoneum and suppresses the stemness and viability of those, that lead to enhanced sensitivity for gemcitabine

**Fig. 7:**
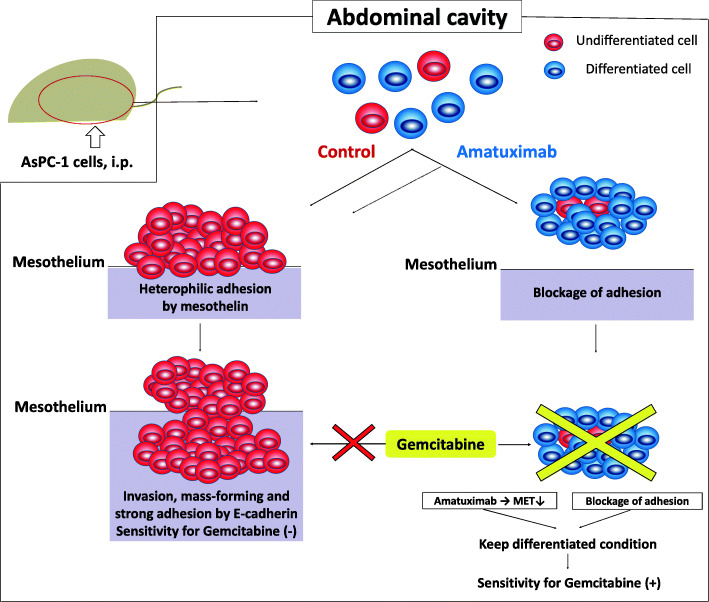
Schematic model for the proposed mechanism by Amatuximab inhibits the chemoresistance of pancreatic cancer in the peritoneal dissemination model. Pancreatic cancer cells disseminated in the abdominal cavity adhere to the peritoneum and dedifferentiate, invade and undergo metastasis. The stable metastases eventually acquire resistance to gemcitabine. Upon treatment with Amatuximab that blocks the adhesion and the molecular change by mesothelin, although a part of disseminated cancer cells makes peritoneal metastasis alike those without Amatuximab, another part of cancer cells was inhibited to adhere to the peritoneum and keep differentiated floating in the ascites. These floating cancer cell clusters have high sensitivity for gemcitabine and can be eliminated by the combination chemotherapy of gemcitabine and Amatuximab

A schematic for a model of our proposed hypothesis is shown in Fig. 7. Pancreatic cancer cells disseminated in the abdominal cavity adhere to the peritoneum and dedifferentiate, invade and undergo metastasis. The stable metastases eventually acquire resistance to gemcitabine. Upon treatment with Amatuximab that blocks the adhesion and the molecular change by mesothelin, although a part of disseminated cancer cells makes peritoneal metastasis alike those without Amatuximab, another part of cancer cells was inhibited to adhere to the peritoneum and keep differentiated floating in the ascites. These floating cancer cell clusters have high sensitivity for gemcitabine and can be eliminated by the combination chemotherapy of gemcitabine and Amatuximab.

## Discussion

In the present study, we demonstrated significant effects of Amatuximab by mesothelin blockage in pancreatic cancer cells. Our in vitro results showed that Amatuximab treatment suppressed the invasiveness and gemcitabine sensitivity of AsPC-1 and Capan-2 pancreatic cancer cells. Amatuximab also induced the downregulation of CSC-related proteins such as pMET in these cell lines.

A full understanding of the biological functions of mesothelin is lacking given that mesothelin knockout mice do not show any developmental phenotype [23]. Recent reports indicate that mesothelin may play an important role in cell adherence, cell survival/proliferation, tumor progression and chemoresistance [24]. In pancreatic cancer, Stat3 plays a pivotal role in oncogenic transformation [25, 26], cell survival, proliferation [25, 27] and resistance to apoptosis [28]. Stat3 is also aberrantly activated in a subset of pancreatic tumor tissues and cell lines [27]. Bharadwaj et al. showed that MSLN upregulation induces the activation of Stat3 in pancreatic cancer cells [29]. Furthermore, the authors showed that mesothelin induced an NFκB/Akt-dependent anti-apoptotic pathway that can protect pancreatic cancer cells from TNF-α-induced apoptosis, and this was a probable mechanism of pancreatic cancer cell survival in midst of inflammation and inflammatory mediators. Other groups found that mesothelin could confer resistance to cytotoxic drug-induced apoptosis via the ERK signaling pathway [30, 31]. Some mesothelin monoclonal antibodies were reported as unable to inhibit cancer cell proliferation because the majority of these antibodies target N terminal region I rather than a key signaling domain in mesothelin. Our results showed that Amatuximab did not inhibit cell proliferation, but the agent suppressed the invasiveness and chemoresistance in AsPC-1 and Capan-2 pancreatic cancer cells. We could observe the slight changes of the molecule expression level in the pathway described above in AsPC-1 only. These results might be caused by the difference of the expression level of mesothelin, that of the expression pattern of MMP7, or that of the mutation pattern of p53 between AsPC-1 cells and Capan-2 cells [32, 33]. These factors might lead to the dissociated results.

CSCs are a population of undifferentiated tumorigenic cells that are responsible for tumor initiation, tumor maintenance and tumor cell spreading to distant organ sites [34]. These cells exhibit unlimited proliferation potential, self-renewal ability and the capacity for the generation of a progeny of differentiated cells which constitute the major tumor population. CSCs can be characterized by chemoresistance [35], multipotency, tumorigenicity [36], stem gene expression [37] and aldehyde dehydrogenase activity [38]. Several studies showed that chemotherapy treatment in pancreatic cancer cell lines led to an increased number of CSCs [39, 40]. CSCs are also thought to be related to the invasiveness of cancer cells [41]. Ortensi et al. reported that brain tumor cells enriched for stem cell markers displayed greater migratory and invasive potential compared with stem cell marker-negative tumor cells. We can identify the CSCs by the expression of CSC-specific cell surface markers, such as CD133, CD44, c-MET and ALDH1 [42]. He et al. showed that mesothelin regulates EMT and CSC traits. Knockdown of mesothelin upregulates epithelial and adhesion molecules on one side and on the other side that downregulates mesenchymal and CSC regulatory genes that relieves self-renewal, proliferation, dissemination and metastasis of cancer cells. Overexpression of mesothelin in normal cells stimulates anchorage-independent growth, migration and invasion [11]. In our study, the mesothelin blockage by Amatuximab directly suppressed the expression level of pMET and led to suppression of malignant features of AsPC-1 and Capan-2 pancreatic cancer cells. To the best of our knowledge, this is the first study to establish a relationship between mesothelin blockage and CSC markers.

MET (also known as c-MET) is a receptor of the tyrosine kinase family that acts as a proto-oncogene and is stimulated by hepatocyte growth factor to mediate motility, invasion, and metastasis [43]. The intracellular signaling cascades activated by MET include the RAS-MAPK and PI3K-Akt pathways, NFκB and Wnt/GSK-3β/β-Catenin signaling [44]. The levels of c-MET are increased in pancreatic carcinoma where c-MET signaling induces growth and invasion and some authors have reported c-MET as a stem cell marker in pancreatic tissue [44]. In our results, we concluded that MET was the important factor in the effects of Amatuximab by mesothelin blockage in pancreatic cancer cells.

Although the molecular changes of AsPC-1 show the limited effects of Amatuximab in vitro study, drastic changes observed in vivo study. Using the peritoneal dissemination model, we demonstrated that several proteins related to proliferation, cancer stemness and chemoresistance were suppressed in the cancer cell clusters (which we named sherbet-like aggregates) that were blocked to attach to peritoneum by mesothelin blockage. We previously demonstrated that the sherbet-like aggregates were sensitive to gemcitabine [21]. Marjanovic presented a plastic CSC theory as a model of tumor heterogeneity [45]. The classical CSC theory proposes that tumor heterogeneity would arise when cancer cells within a given tumor reside in different states of stemness or differentiation. Critical to this theory is the notion that CSC-to-non-CSC conversion is a unidirectional process. The plastic CSC theory describes an evolving model in which bidirectional conversions exist between non-CSCs and CSCs that are controlled by extrinsic features, such as extracellular matrix or blood vessels, and intrinsic features, such as genetic or epigenetic changes. This model implies that non-CSCs can continually create CSC populations throughout tumorigenesis. In other words, this theory suggests that CSCs are kept in differentiated states by their surroundings, and their malignancy is suppressed in this condition. Stankevicius et al. reported the importance of microenvironment, including scaffold, for the cancer cell stemness in human colorectal cancer cells [46]. The authors demonstrated that the expressions of several CSC markers were increased in 3D cultures considered as scaffold compared with those in 2D monolayer cultures. Our results suggested that not only the direct effects of Amatuximab but also the environmental changes induced by Amatuximab could control the stemness of pancreatic cancer cells expressing high mesothelin, and these resulted in an improvement of sensitivity for gemcitabine in mesothelin-expressing pancreatic cancer cells.

There are several limitations in the present study. First, we did not demonstrate the relationship between the expression of mesothelin and CSCs. We need to examine the expression changes of CSC molecules using the cells those expression of mesothelin were genetically controlled. Second, we could not confirm the definite role of p-MET downregulation in the antitumor activity of Amatuximab directly in this study. To confirm that, we should carry out the experiment such as invasion assay, migration assay and gemcitabine sensitivity experiments using AsPC-1 cells or Capan-2 cells those expression of p-MET is controlled genetically or using inhibitor for p-MET. Third, we carried out our experiments using whole pancreatic cancer cells, not dividing cells to CSCs or non-CSCs. We need to determine the specific effects of Amatuximab on CSC population separated by sorting technique for further investigation in the future. Fourth, our in vitro data could not show the relationship between the inhibition of invasion/migration and enhancement of antitumor activity of gemcitabine in mesothelin-high pancreatic cancer cell lines. The setting of the in vitro experiments could not mimic the condition of ex vivo study completely. 3D culture might be suitable for the investigation of cancer stemness and of our ex vivo study. Shishido reported the relationship between the ovarian cancer cells and peritoneal cells in the co-culture experiments [47]. The ovarian cancer cells showed the stem like features more strongly in co-culture condition than in monoculture. Co-culture study might be useful in our investigation of CSCs. Fifth, our data could not reveal how Amatuximab caused the generations of sherbet like aggregates. Some previous reports showed that CA125 was expressed in the peritoneum in the mouse [48] and that Amatuximab inhibited the interaction of mesothelin-CA125 [49]. Although we tried to reveal the direct evidence showing that Amatuximab inhibited the adhesion of cancer cells to peritoneum, we could not confirm the mechanism in this study. Additionally, we examined the in vivo experiments using only AsPC-1 cell lines. To validate the efficacy of Amatuximab for pancreatic cancer cells and the relationship between effect of Amatuximab and expression of mesothelin, we need to perform the experiment using other several pancreatic cancer cell lines. Our findings are limited to pancreatic cancer cell lines. Further research is needed in other types of cancer cells that highly express mesothelin. Finally, we did not demonstrate the relationship between the microenvironment and the phenomenon in the previous study. We need to reveal these mechanisms by establishing the experimental system in those the microenvironment was controlled.

## Conclusion

In summary, we demonstrated that mesothelin blockage by Amatuximab directly suppressed the expression of CSC-related molecules and cell invasiveness. In addition, mesothelin blockage suppressed the adhesion of pancreatic cancer cells to mesothelium in a peritoneal dissemination mouse model. These effects led to the enhancement of sensitivity for gemcitabine. These results suggest a new possibility for Amatuximab as a therapeutic agent for mesothelin-expressing cancers. Future studies will examine these findings through in vivo experiments and clinical investigations.

## Supplementary Information

**Additional file 1: Supplemental Table 1.** First antibodies those were used for western blotting analysis and immunohistochemistry.

**Additional file 2: Supplemental Table 2.** The primers list those were used for Quantitative RT-PCR.

**Additional file 3: Supplemental Figure 1.** Analysis for mesothelin expression in the four human pancreatic cancer cells by western blotting using another primary antibody. Ful-length blots are presented in Supplementary Figure 6. Densitometric analysis of western blots was performed using a ChemiDoc XRS Plus system with Image Lab Software (Bio-Rad, Hercules, CA, USA). We cut the membranes according to the standard protein size markers and detected the blot using the images in those the blotting picture and marker were merged.

**Additional file 4: Supplemental Figure2.** Corresponding uncropped full-length blot images for Fig. 1a. The cropped blots were marked with black frame. Densitometric analysis of western blots was performed using a ChemiDoc XRS Plus system with Image Lab Software (Bio-Rad, Hercules, CA, USA). We cut the membranes according to the standard protein size markers and detected the blot using the images in those the blotting picture and marker were merged.

**Additional file 5: Supplemental Figure 3.** Corresponding uncropped full-length blot images for Fig. 3a. The cropped blots were marked with black frame. Densitometric analysis of western blots was performed using a ChemiDoc XRS Plus system with Image Lab Software (Bio-Rad, Hercules, CA, USA). We cut the membranes according to the standard protein size markers and detected the blot using the images in those the blotting picture and marker were merged.

**Additional file 6: Supplemental Figure 4.** Corresponding uncropped full-length blot images for Fig. 3b. The cropped blots were marked with black frame. Densitometric analysis of western blots was performed using a ChemiDoc XRS Plus system with Image Lab Software (Bio-Rad, Hercules, CA, USA). We cut the membranes according to the standard protein size markers and detected the blot using the images in those the blotting picture and marker were merged.

**Additional file 7: Supplemental Figure 5.** Corresponding uncropped full-length blot images for Fig. 3c. The cropped blots were marked with black frame. Densitometric analysis of western blots was performed using a ChemiDoc XRS Plus system with Image Lab Software (Bio-Rad, Hercules, CA, USA). We cut the membranes according to the standard protein size markers and detected the blot using the images in those the blotting picture and marker were merged.

**Additional file 8: Supplemental Figure 6.** Corresponding uncropped full-length blot images for supplemental Figure 1. The cropped blots were marked with black frame. Densitometric analysis of western blots was performed using a ChemiDoc XRS Plus system with Image Lab Software (Bio-Rad, Hercules, CA, USA). We cut the membranes according to the standard protein size markers and detected the blot using the images in those the blotting picture and marker were merged.

**Additional file 9: Supplemental Figure 7.** Analysis of mesothelin expression in the four human pancreatic cancer cells by immunocytochemistry: (a) AsPC-1, (b) Capan-2, (c) Panc-1 and (d) MIA Paca-2 cells. The image of Capan-2 was taken by deferent researcher in another time, so in a little bit deferent condition. Scale bar, 100 μm.

## Data Availability

All data generated or analysed during this study are included in this published article and its supplementary information files and available.

## Abbreviations

IgG: Immunogloblin G
pMET: Phosphorylated Met
ALDH1: Aldehyde dehydrogenase 1
CD44: Cluster of differentiation 44
cMET: Hepatocyte growth factor receptor
EMT: Epithelial-Mesenchymal Transition
CSC: Cancer stem cell
CA125: Carbohydrate antigen 125
EDTA: Ethylenediaminetetraacetic acid
FBS: Fetal bovine serum
FACS: Fluorescence activated cell sorter
RT-PCR: Reverse transcriptase polymerase chain reaction
GAPDH: Glyceraldehyde 3-phosphate dehydrogenase
mRNA: Messenger ribonucleic acid
ERK: Extracellular signal-regulated Kinase
MEK: MAPK/ERK kinase
NFκB: Nuclear factor-kappa B
TNF-α: Tumor Necrosis Factor α
MMP7: Matrix metalloproteinase
CD133: Cluster of differentiation 133
MAPK: Mitogen-activated Protein Kinase
PI3K: Phosphoinositide 3-kinase
GSK-3β: Glycogen synthase kinase 3β

## References

[1] Kleeff J, Korc M, Apte M, La Vecchia C, Johnson CD, Biankin AV, Neale RE, Tempero M, Tuveson DA, Hruban RH (2016). Pancreatic cancer. Nat Rev Dis Primers.

[2] Tempero MA, Malafa MP, Al-Hawary M, Asbun H, Bain A, Behrman SW, Benson AB, Binder E, Cardin DB, Cha C (2017). Pancreatic adenocarcinoma, version 2.2017, NCCN clinical practice guidelines in oncology. J Natl Compr Canc Netw.

[3] Vauthey JN, Dixon E (2009). AHPBA/SSO/SSAT consensus conference on Resectable and borderline Resectable pancreatic Cancer: rationale and overview of the conference. Ann Surg Oncol.

[4] Hattangadi JA, Hong TS, Yeap BY, Mamon HJ (2009). Results and patterns of failure in patients treated with adjuvant combined chemoradiation therapy for resected pancreatic adenocarcinoma. Cancer.

[5] Neoptolemos JP, Stocken DD, Bassi C, Ghaneh P, Cunningham D, Goldstein D, Padbury R, Moore MJ, Gallinger S, Mariette C (2010). Adjuvant chemotherapy with fluorouracil plus folinic acid vs gemcitabine following pancreatic cancer resection: a randomized controlled trial. JAMA.

[6] Uesaka K, Boku N, Fukutomi A, Okamura Y, Konishi M, Matsumoto I, Kaneoka Y, Shimizu Y, Nakamori S, Sakamoto H (2016). Adjuvant chemotherapy of S-1 versus gemcitabine for resected pancreatic cancer: a phase 3, open-label, randomised, non-inferiority trial (JASPAC 01). Lancet (London, England).

[7] Ducreux M, Boige V, Malka D (2007). Treatment of advanced pancreatic cancer. Semin Oncol.

[8] Chang K, Pastan I (1996). Molecular cloning of mesothelin, a differentiation antigen present on mesothelium, mesotheliomas, and ovarian cancers. Proc Natl Acad Sci U S A.

[9] Hassan R, Laszik ZG, Lerner M, Raffeld M, Postier R, Brackett D (2005). Mesothelin is overexpressed in pancreaticobiliary adenocarcinomas but not in normal pancreas and chronic pancreatitis. Am J Clin Pathol.

[10] Li M, Bharadwaj U, Zhang R, Zhang S, Mu H, Fisher WE, Brunicardi FC, Chen C, Yao Q (2008). Mesothelin is a malignant factor and therapeutic vaccine target for pancreatic cancer. Mol Cancer Ther.

[11] He X, Wang L, Riedel H, Wang K, Yang Y, Dinu CZ, Rojanasakul Y (2017). Mesothelin promotes epithelial-to-mesenchymal transition and tumorigenicity of human lung cancer and mesothelioma cells. Mol Cancer.

[12] Einama T, Homma S, Kamachi H, Kawamata F, Takahashi K, Takahashi N, Taniguchi M, Kamiyama T, Furukawa H, Matsuno Y (2012). Luminal membrane expression of mesothelin is a prominent poor prognostic factor for gastric cancer. Br J Cancer.

[13] Einama T, Kamachi H, Nishihara H, Homma S, Kanno H, Ishikawa M, Kawamata F, Konishi Y, Sato M, Tahara M (2015). Importance of luminal membrane mesothelin expression in intraductal papillary mucinous neoplasms. Oncol Lett.

[14] Einama T, Kamachi H, Nishihara H, Homma S, Kanno H, Takahashi K, Sasaki A, Tahara M, Okada K, Muraoka S (2011). Co-expression of mesothelin and CA125 correlates with unfavorable patient outcome in pancreatic ductal adenocarcinoma. Pancreas.

[15] Einama T, Kawamata F, Kamachi H, Nishihara H, Homma S, Matsuzawa F, Mizukami T, Konishi Y, Tahara M, Kamiyama T (2016). Clinical impacts of mesothelin expression in gastrointestinal carcinomas. World J Gastrointest Pathophysiol.

[16] Kawamata F, Homma S, Kamachi H, Einama T, Kato Y, Tsuda M, Tanaka S, Maeda M, Kajino K, Hino O (2014). C-ERC/mesothelin provokes lymphatic invasion of colorectal adenocarcinoma. J Gastroenterol.

[17] Kawamata F, Kamachi H, Einama T, Homma S, Tahara M, Miyazaki M, Tanaka S, Kamiyama T, Nishihara H, Taketomi A (2012). Intracellular localization of mesothelin predicts patient prognosis of extrahepatic bile duct cancer. Int J Oncol.

[18] Morello A, Sadelain M, Adusumilli PS (2016). Mesothelin-targeted CARs: driving T cells to solid tumors. Cancer Discov.

[19] Hassan R, Ebel W, Routhier EL, Patel R, Kline JB, Zhang J, Chao Q, Jacob S, Turchin H, Gibbs L (2007). Preclinical evaluation of MORAb-009, a chimeric antibody targeting tumor-associated mesothelin. Cancer Immun.

[20] Fujisaka Y, Kurata T, Tanaka K, Kudo T, Okamoto K, Tsurutani J, Kaneda H, Okamoto I, Namiki M, Kitamura C (2015). Phase I study of amatuximab, a novel monoclonal antibody to mesothelin, in Japanese patients with advanced solid tumors. Investig New Drugs.

[21] Mizukami T, Kamachi H, Fujii Y, Matsuzawa F, Einama T, Kawamata F, Kobayashi N, Hatanaka Y, Taketomi A (2018). The anti-mesothelin monoclonal antibody amatuximab enhances the anti-tumor effect of gemcitabine against mesothelin-high expressing pancreatic cancer cells in a peritoneal metastasis mouse model. Oncotarget.

[22] Awasthi N, Monahan S, Stefaniak A, Schwarz MA, Schwarz RE (2018). Inhibition of the MEK/ERK pathway augments nab-paclitaxel-based chemotherapy effects in preclinical models of pancreatic cancer. Oncotarget.

[23] Bera TK, Pastan I (2000). Mesothelin is not required for normal mouse development or reproduction. Mol Cell Biol.

[24] Tang Z, Qian M, Ho M (2013). The role of mesothelin in tumor progression and targeted therapy. Anti Cancer Agents Med Chem.

[25] Scholz A, Heinze S, Detjen KM, Peters M, Welzel M, Hauff P, Schirner M, Wiedenmann B, Rosewicz S (2003). Activated signal transducer and activator of transcription 3 (STAT3) supports the malignant phenotype of human pancreatic cancer. Gastroenterology.

[26] DeArmond D, Brattain MG, Jessup JM, Kreisberg J, Malik S, Zhao S, Freeman JW (2003). Autocrine-mediated ErbB-2 kinase activation of STAT3 is required for growth factor independence of pancreatic cancer cell lines. Oncogene.

[27] Toyonaga T, Nakano K, Nagano M, Zhao G, Yamaguchi K, Kuroki S, Eguchi T, Chijiiwa K, Tsuneyoshi M, Tanaka M (2003). Blockade of constitutively activated Janus kinase/signal transducer and activator of transcription-3 pathway inhibits growth of human pancreatic cancer. Cancer Lett.

[28] Greten FR, Weber CK, Greten TF, Schneider G, Wagner M, Adler G, Schmid RM (2002). Stat3 and NF-kappaB activation prevents apoptosis in pancreatic carcinogenesis. Gastroenterology.

[29] Bharadwaj U, Li M, Chen C, Yao Q (2008). Mesothelin-induced pancreatic cancer cell proliferation involves alteration of cyclin E via activation of signal transducer and activator of transcription protein 3. Mol Cancer Res.

[30] Uehara N, Matsuoka Y, Tsubura A (2008). Mesothelin promotes anchorage-independent growth and prevents anoikis via extracellular signal-regulated kinase signaling pathway in human breast cancer cells. Mol Cancer Res.

[31] Chang MC, Chen CA, Hsieh CY, Lee CN, Su YN, Hu YH, Cheng WF (2009). Mesothelin inhibits paclitaxel-induced apoptosis through the PI3K pathway. Biochem J.

[32] Tan X, Egami H, Abe M, Nozawa F, Hirota M, Ogawa M (2005). Involvement of MMP-7 in invasion of pancreatic cancer cells through activation of the EGFR mediated MEK-ERK signal transduction pathway. J Clin Pathol.

[33] Zheng C, Jia W, Tang Y, Zhao H, Jiang Y, Sun S (2012). Mesothelin regulates growth and apoptosis in pancreatic cancer cells through p53-dependent and -independent signal pathway. J Exp Clin Cancer Res.

[34] Reya T, Morrison SJ, Clarke MF, Weissman IL (2001). Stem cells, cancer, and cancer stem cells. Nature.

[35] Dean M, Fojo T, Bates S (2005). Tumour stem cells and drug resistance. Nat Rev Cancer.

[36] Rosen JM, Jordan CT (2009). The increasing complexity of the cancer stem cell paradigm. Science.

[37] Ouyang G, Wang Z, Fang X, Liu J, Yang CJ (2010). Molecular signaling of the epithelial to mesenchymal transition in generating and maintaining cancer stem cells. Cell Mol Life Sci.

[38] Awad O, Yustein JT, Shah P, Gul N, Katuri V, O'Neill A, Kong Y, Brown ML, Toretsky JA, Loeb DM (2010). High ALDH activity identifies chemotherapy-resistant Ewing's sarcoma stem cells that retain sensitivity to EWS-FLI1 inhibition. PLoS One.

[39] Mueller MT, Hermann PC, Witthauer J, Rubio-Viqueira B, Leicht SF, Huber S, Ellwart JW, Mustafa M, Bartenstein P, D'Haese JG (2009). Combined targeted treatment to eliminate tumorigenic cancer stem cells in human pancreatic cancer. Gastroenterology.

[40] Dylla SJ, Beviglia L, Park IK, Chartier C, Raval J, Ngan L, Pickell K, Aguilar J, Lazetic S, Smith-Berdan S (2008). Colorectal cancer stem cells are enriched in xenogeneic tumors following chemotherapy. PLoS One.

[41] Ortensi B, Setti M, Osti D, Pelicci G (2013). Cancer stem cell contribution to glioblastoma invasiveness. Stem Cell Res Ther.

[42] Eramo A, Lotti F, Sette G, Pilozzi E, Biffoni M, Di Virgilio A, Conticello C, Ruco L, Peschle C, De Maria R (2008). Identification and expansion of the tumorigenic lung cancer stem cell population. Cell Death Differ.

[43] Michieli P, Mazzone M, Basilico C, Cavassa S, Sottile A, Naldini L, Comoglio PM (2004). Targeting the tumor and its microenvironment by a dual-function decoy met receptor. Cancer Cell.

[44] Gherardi E, Birchmeier W, Birchmeier C, Vande Woude G (2012). Targeting MET in cancer: rationale and progress. Nat Rev Cancer.

[45] Marjanovic ND, Weinberg RA, Chaffer CL (2013). Cell plasticity and heterogeneity in cancer. Clin Chem.

[46] Stankevicius V, Kunigenas L, Stankunas E, Kuodyte K, Strainiene E, Cicenas J, Samalavicius NE, Suziedelis K (2017). The expression of cancer stem cell markers in human colorectal carcinoma cells in a microenvironment dependent manner. Biochem Biophys Res Commun.

[47] Shishido A, Mori S, Yokoyama Y, Hamada Y, Minami K, Qian Y, Wang J, Hirose H, Wu X, Kawaguchi N (2018). Mesothelial cells facilitate cancer stem-like properties in spheroids of ovarian cancer cells. Oncol Rep.

[48] Wang Y, Cheon DJ, Lu Z, Cunningham SL, Chen CM, Luo RZ, Xing D, Orsulic S, Bast RC, Behringer RR (2008). MUC16 expression during embryogenesis, in adult tissues, and ovarian cancer in the mouse. Differentiation.

[49] Hassan R, Schweizer C, Lu KF, Schuler B, Remaley AT, Weil SC, Pastan I (2010). Inhibition of mesothelin-CA-125 interaction in patients with mesothelioma by the anti-mesothelin monoclonal antibody MORAb-009: Implications for cancer therapy. Lung Cancer.

