# Supplementary material for: Mesothelin blockage by Amatuximab suppresses cell invasiveness, enhances gemcitabine sensitivity and regulates cancer cell stemness in mesothelin-positive pancreatic cancer cells
Source: BMC Cancer. 2021 Feb 26;21:200. doi: 10.1186/s12885-020-07722-3 (PMC7912898; doi:10.1186/s12885-020-07722-3)
Supplement: Supplementary file 1 — Additional file 1: Supplemental Table 1. First antibodies those were used for western blotting analysis and immunohistochemistry. [file 12885_2020_7722_MOESM1_ESM.docx]

Supplemental Table 1: First antibodies those were used for western blotting analysis and immunohistochemistry

| Antibody name | Product code and manufacturer | Antibody dilution in experiments |
| --- | --- | --- |
| Mesothelin rabbit polyclonal | #TA308767, ORIGENE | WB 1:1000 |
| Mesothelin mouse monoclonal | #10357, 22A31, Immuno-Biological Laboratories | WB 1:2000; IHC 1: 50 |
| ALDH1 mouse monoclonal | #611195, 44/ALDH, BD Biosciences | WB 1:2000; IHC 1: 100 |
| CD44 mouse monoclonal | #3570, 156-3C11, Cell Signaling Technology | WB 1:2000; IHC 1: 50 |
| p-MET rabbit monoclonal | #3077, D26, Cell signaling Technology | WB 1:2000 |
| E-Cadherin rabbit monoclonal | #3195, 24E10, Cell Signaling Technology | WB 1:2000; IHC 1: 50 |
| N-Cadherin rabbit polyclonal | ab18203, Abcam | WB 1:2000; IHC 1: 25 |
| ZO-1 rabbit monoclonal | #8193, D7D12, Cell Signaling Technology | WB 1:2000 |
| GAPDH rabbit monoclonal | #5174, D16H11, Cell Signaling Technology | WB 1:2000 |
| β-actin rabbit monoclonal | #4970, 13E5, Cell Signaling Technology | WB 1:2000 |
| MEK 1/2 rabbit monoclonal | #8727, D1A5, Cell Signaling Technology | WB 1:2000 |
| ERK 1/2 rabbit monoclonal | #4695, 137F5, Cell Signaling Technology | WB 1:2000 |
| p-NFκB rabbit monoclonal | #3033, 93H1, Cell Signaling Technology | WB 1:2000 |
| NFκB rabbit monoclonal | #8242, D14E12, Cell Signaling Technology | WB 1:2000 |
| Vimentin rabbit monoclonal | #5741, D21H3, Cell Signaling Technology | WB 1:2000; IHC 1: 50 |
| p-Stat3 rabbit monoclonal | #9145, D3A7, Cell Signaling Technology | WB 1:2000 |
| Stat3 rabbit monoclonal | #12640, D3Z2G, Cell Signaling Technology | WB 1:2000 |
| mesothelin mouse monoclonal | #MESO-L-CE, clone 5B2, Novocastra | IHC 1: 100 |
| Ki-67 mouse monoclonal | #M7240, MIB-1, Dako | IHC 1: 200 |
| p-ERK 1/2 rabbit monoclonal | #4370, D13.14.4E, Cell Signaling Technology | IHC 1: 400 |
| c-MET mouse monoclonal | M3580, SP58, Spring Bioscience | IHC 1:50 |
| CXCR4 mouse monoclonal | #35-8800, 12G5, Thermo Fisher Scientific | IHC 1:50 |
| CD133 rabbit polyclonal | #PAB12663, Abnova | IHC 1:50 |

WB: Western blot; IHC: Immunohistochemistry
