# Supplementary material for: Mesothelin blockage by Amatuximab suppresses cell invasiveness, enhances gemcitabine sensitivity and regulates cancer cell stemness in mesothelin-positive pancreatic cancer cells
Source: BMC Cancer. 2021 Feb 26;21:200. doi: 10.1186/s12885-020-07722-3 (PMC7912898; doi:10.1186/s12885-020-07722-3)
Supplement: Supplementary file 2 — Additional file 2: Supplemental Table 2. The primers list those were used for Quantitative RT-PCR. [file 12885_2020_7722_MOESM2_ESM.docx]

Supplemental table 2

The following primers were used:

ALDH1 forward, 5’- AAATCCTCTGACCCCAGGAG -3’;

ALDH1 reverse, 5’- GTTTGGCCCCTTCTTTCTTC -3’;

c-MET forward, 5’- AGTGAAGTGGATGGCTTTGG -3’;

c-MET reverse, 5’- ATAAGGTGGGGCTCCTCTTG -3’;

CD44 forward, 5’- GAAAGGAGCAGCACTTCAGG -3’;

CD44 reverse, 5’- GGGTGGAATGTGTCTTGGTC -3’;

E-Cadherin forward, 5’- GACTCGTAACGACGTTGCAC -3’;

E-Cadherin reverse, 5’- GGTCAGTATCAGCCGCTTTC -3’;

GAPDH forward, 5’- ATCAAGTGGGGCGATGCTG -3’;

GAPDH reverse, 5’- ACCCATGACGAACATGGGG -3’.
