# Supplementary material for: Mesothelin blockage by Amatuximab suppresses cell invasiveness, enhances gemcitabine sensitivity and regulates cancer cell stemness in mesothelin-positive pancreatic cancer cells
Source: BMC Cancer. 2021 Feb 26;21:200. doi: 10.1186/s12885-020-07722-3 (PMC7912898; doi:10.1186/s12885-020-07722-3)

Supplemental figure 1

Western blotting analysis of mesothelin expression detected by another primary antibody in human pancreatic cancer cell lines

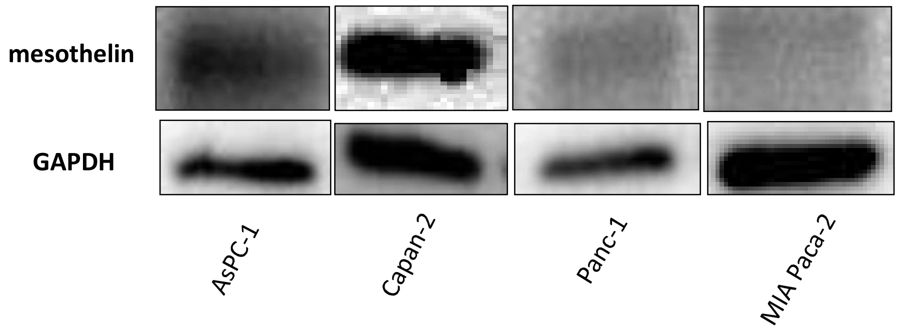

Supplement: Supplementary file 3 — Additional file 3: Supplemental Figure 1. Analysis for mesothelin expression in the four human pancreatic cancer cells by western blotting using another primary antibody. Ful-length blots are presented in Supplementary Figure 6. Densitometric analysis of western blots was performed using a ChemiDoc XRS Plus system with Image Lab Software (Bio-Rad, Hercules, CA, USA). We cut the membranes according to the standard protein size markers and detected the blot using the images in those the blotting picture and marker were merged. [file 12885_2020_7722_MOESM3_ESM.pdf]
