# Supplementary figures and images for: Mesothelin blockage by Amatuximab suppresses cell invasiveness, enhances gemcitabine sensitivity and regulates cancer cell stemness in mesothelin-positive pancreatic cancer cells
Source: BMC Cancer. 2021 Feb 26;21:200. doi: 10.1186/s12885-020-07722-3 (PMC7912898; doi:10.1186/s12885-020-07722-3)

Figure 1A uncropped images

mesothelin

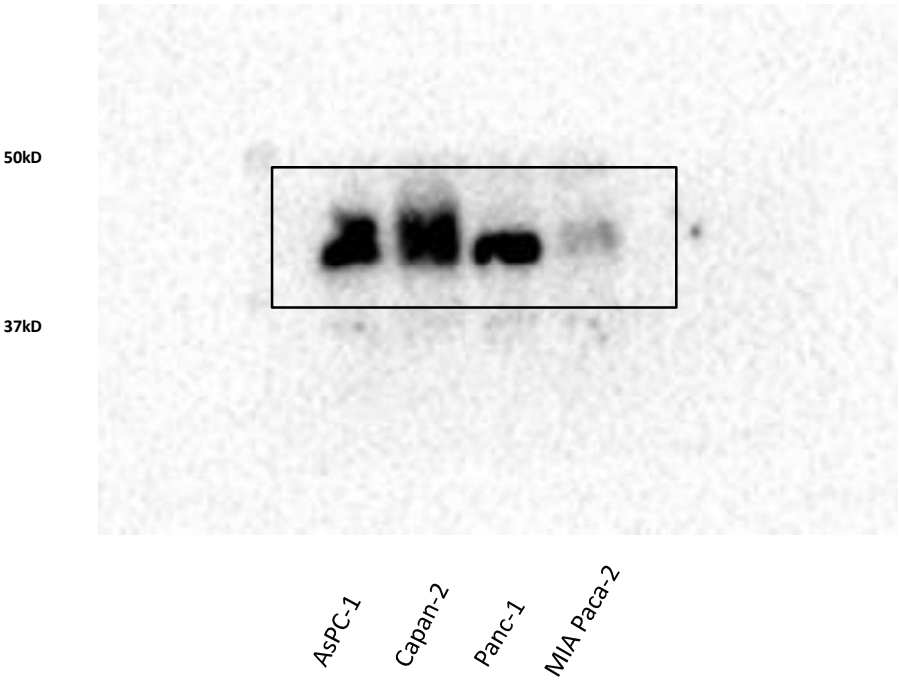

B-actin

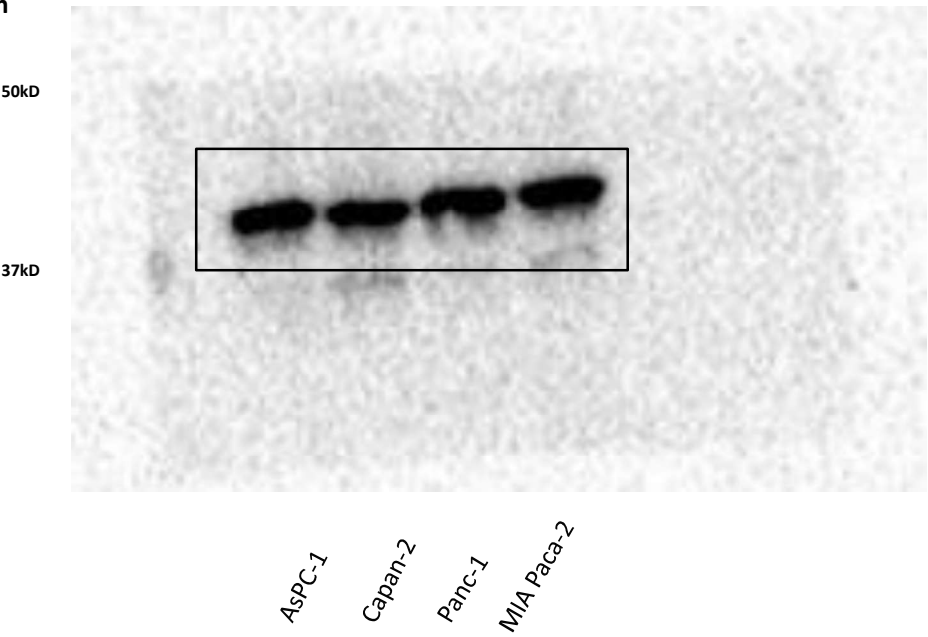

Supplement: Supplementary file 4 — Additional file 4: Supplemental Figure2. Corresponding uncropped full-length blot images for Fig. 1a. The cropped blots were marked with black frame. Densitometric analysis of western blots was performed using a ChemiDoc XRS Plus system with Image Lab Software (Bio-Rad, Hercules, CA, USA). We cut the membranes according to the standard protein size markers and detected the blot using the images in those the blotting picture and marker were merged. [file 12885_2020_7722_MOESM4_ESM.pdf]

Supplemental figure 3

Figure 3A uncropped images

GAPDH

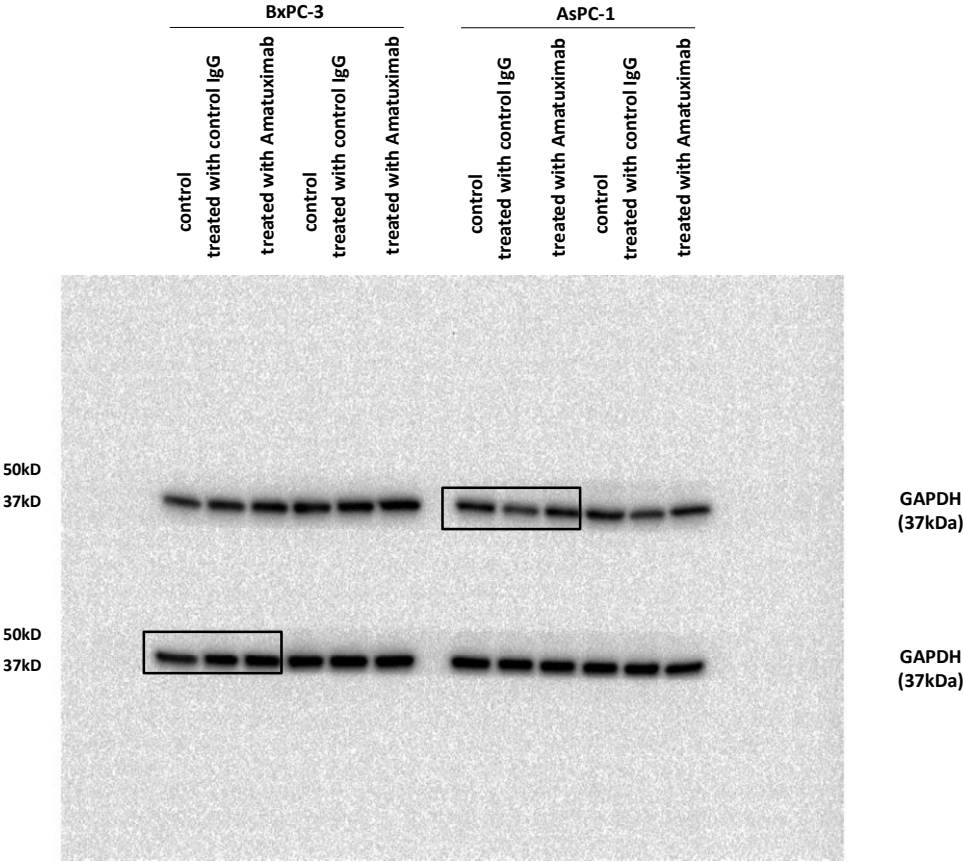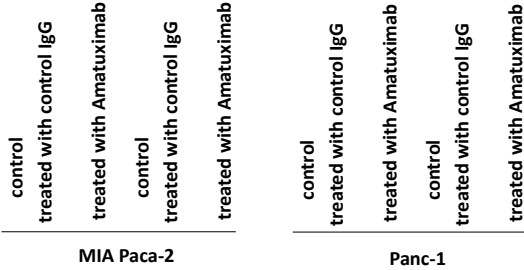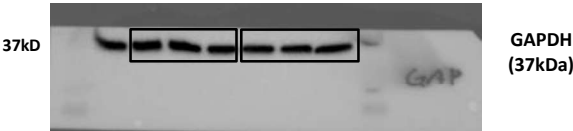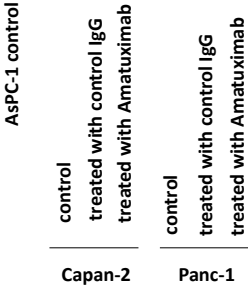

## ALDH1

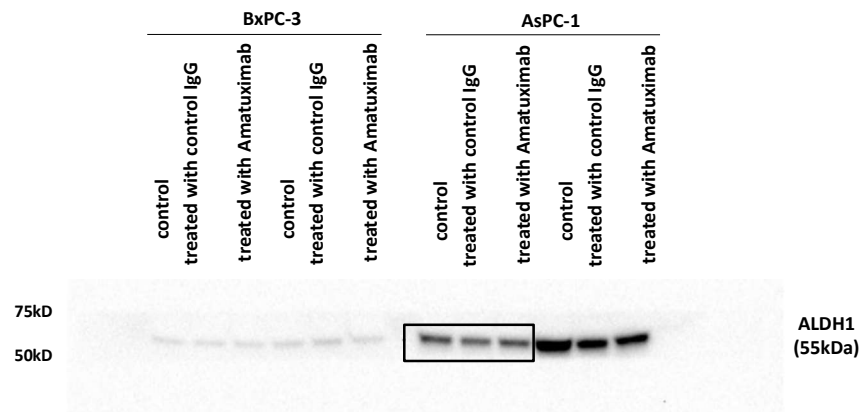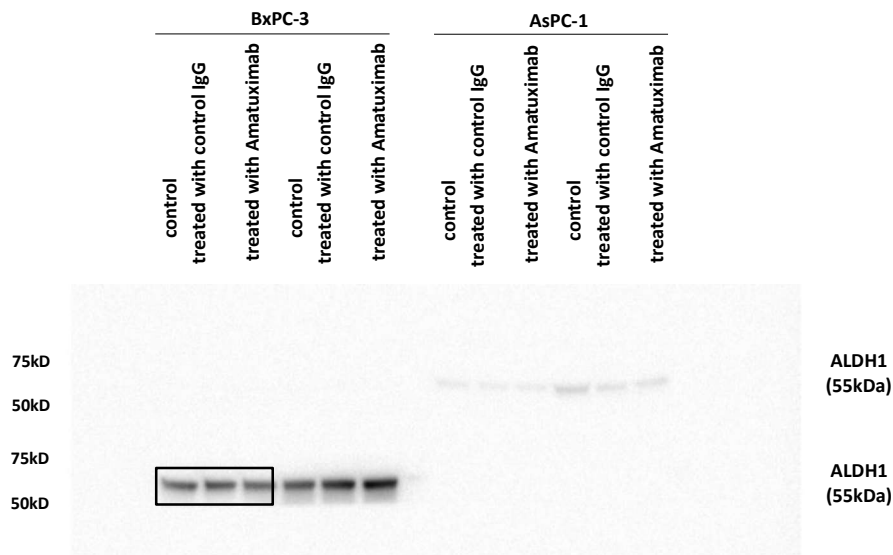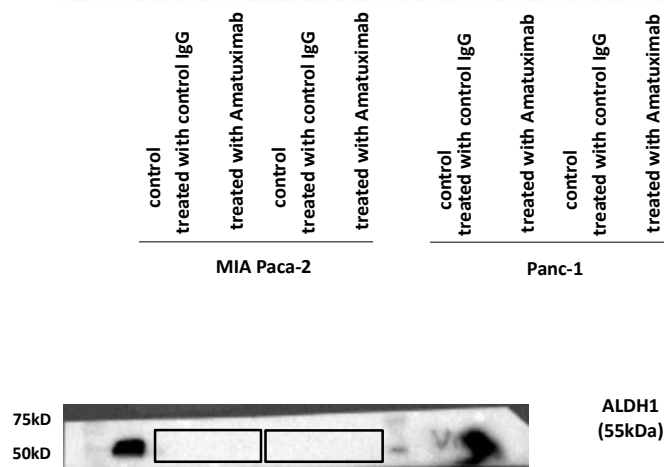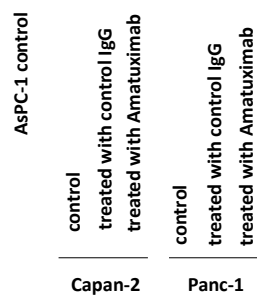

p-MET

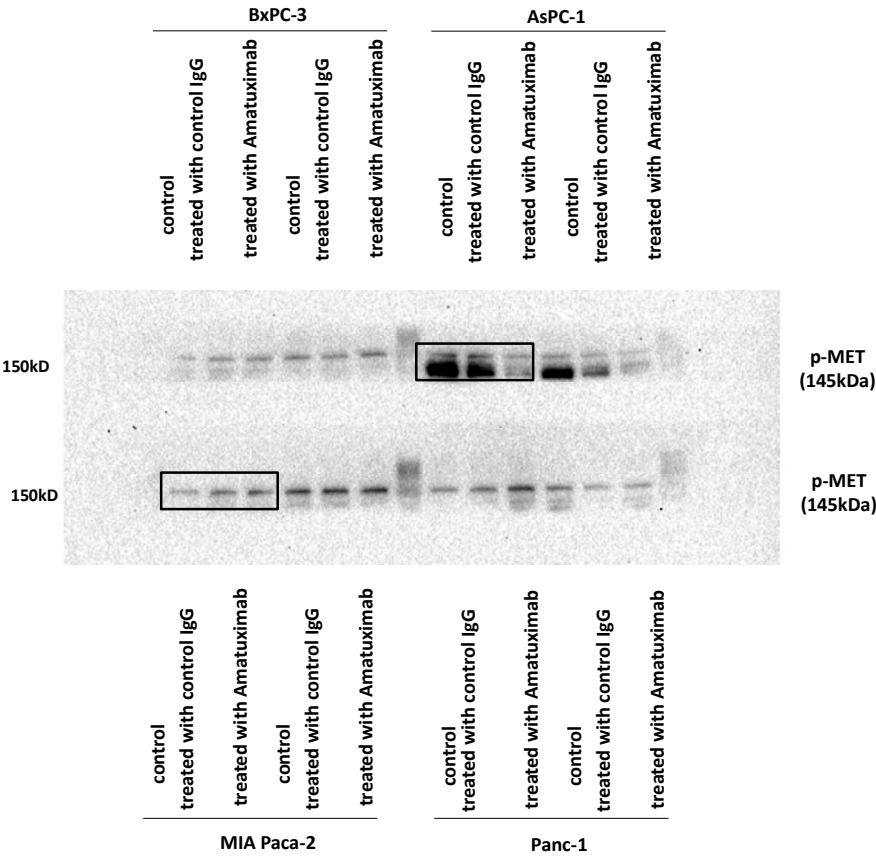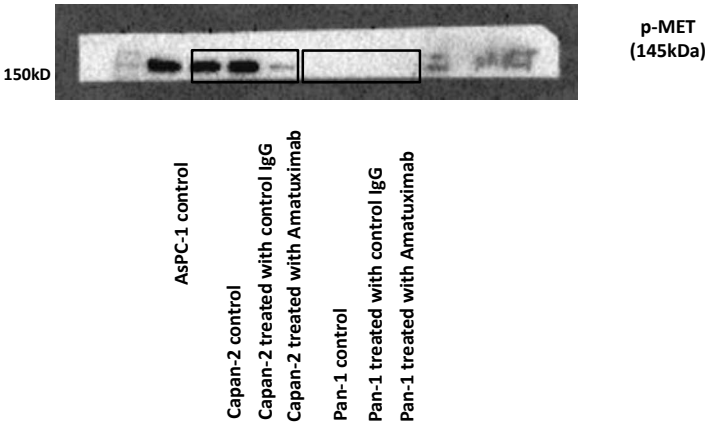

CD44

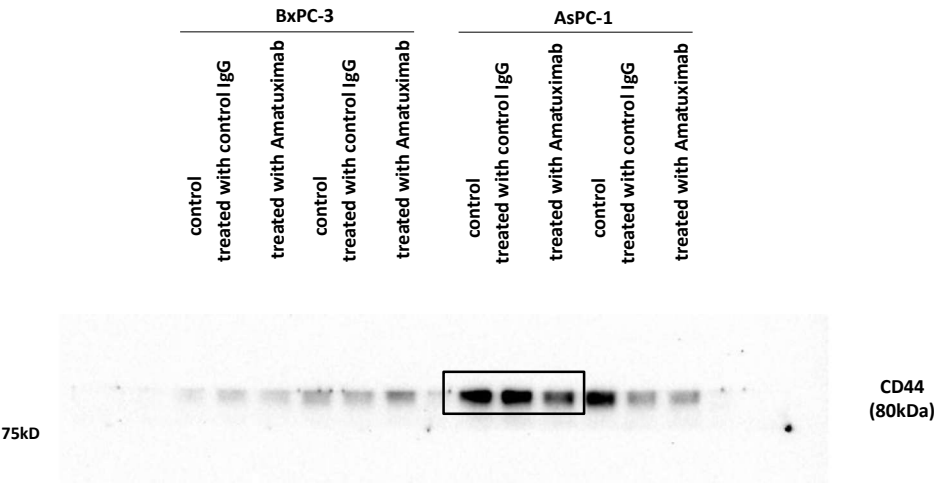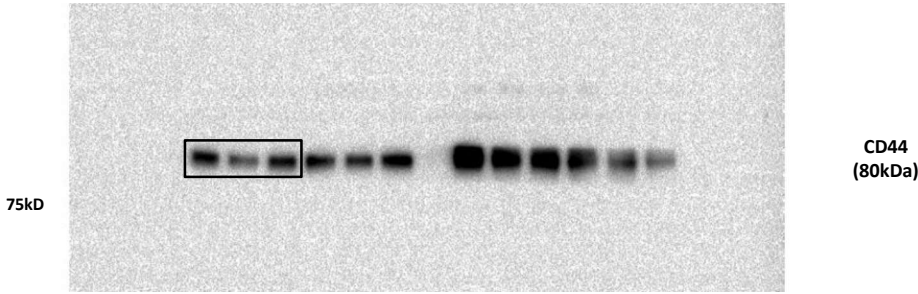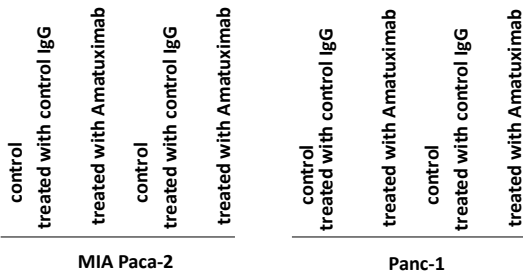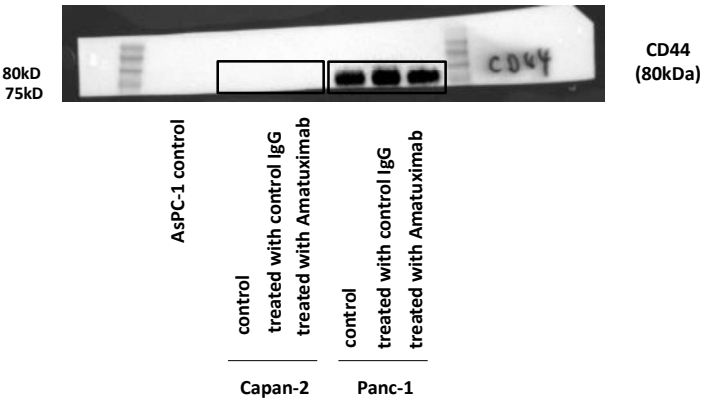

Supplement: Supplementary file 5 — Additional file 5: Supplemental Figure 3. Corresponding uncropped full-length blot images for Fig. 3a. The cropped blots were marked with black frame. Densitometric analysis of western blots was performed using a ChemiDoc XRS Plus system with Image Lab Software (Bio-Rad, Hercules, CA, USA). We cut the membranes according to the standard protein size markers and detected the blot using the images in those the blotting picture and marker were merged. [file 12885_2020_7722_MOESM5_ESM.pdf]

Supplemental figure 4

Figure 3B uncropped images

MEK 1/2

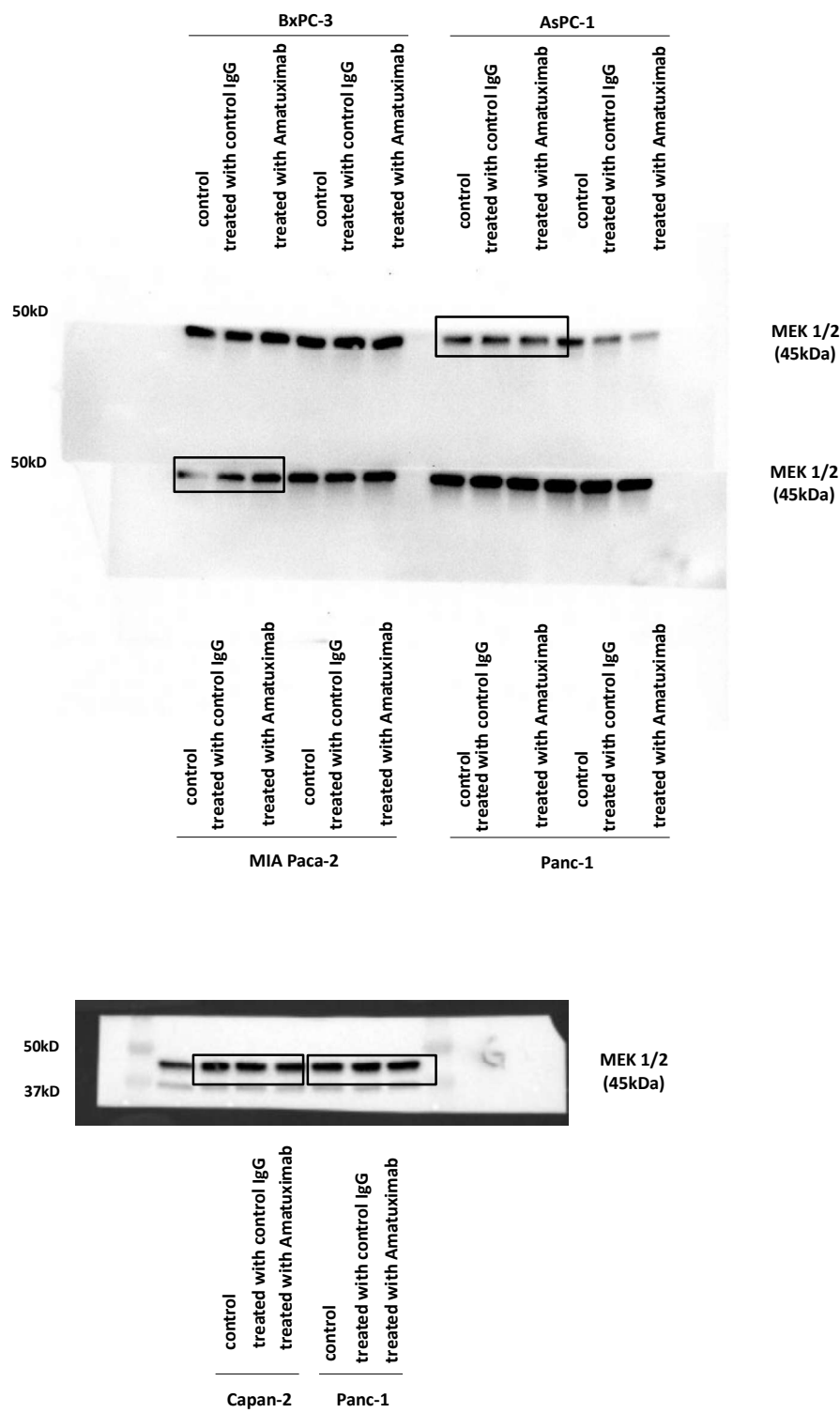

ERK 1/2

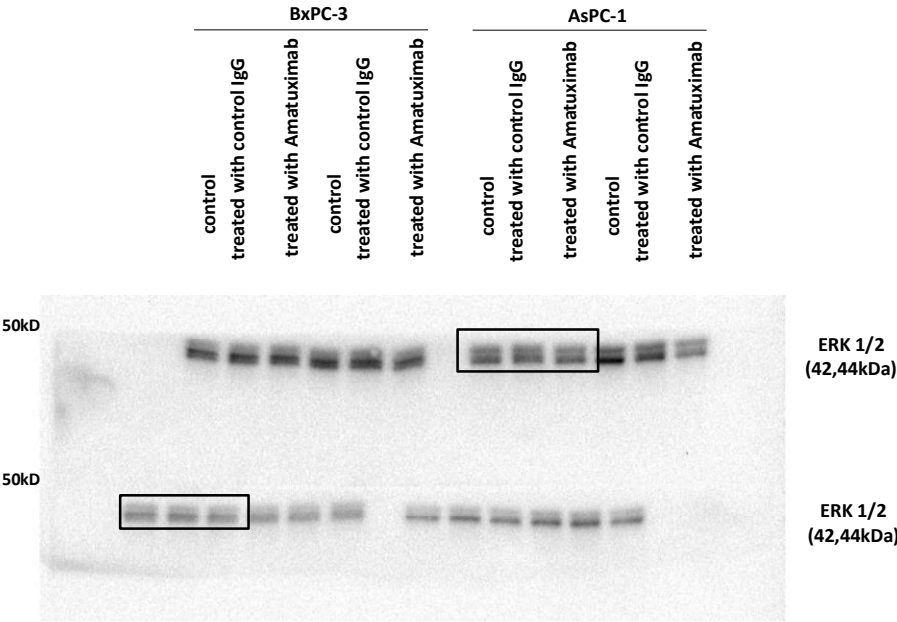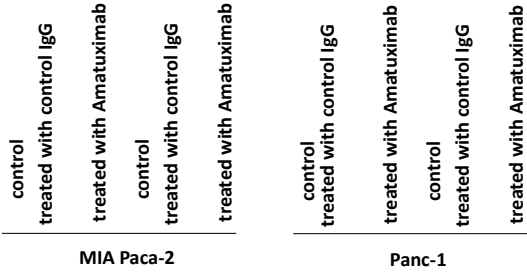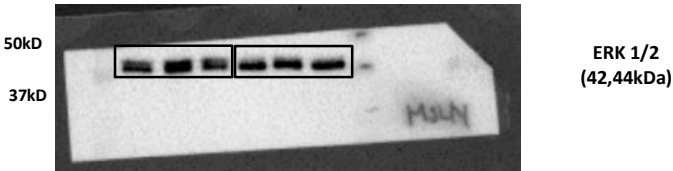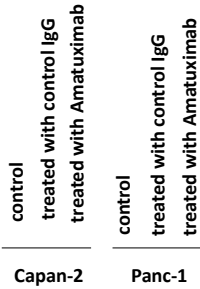

p-NKκB

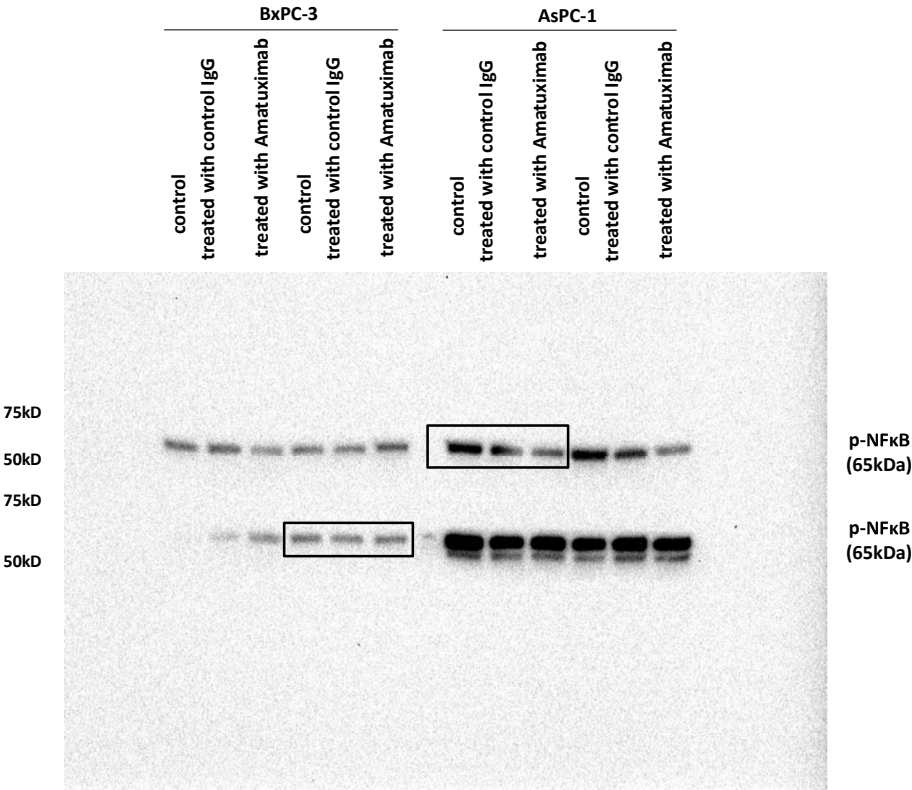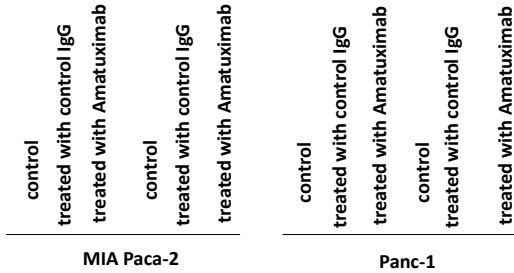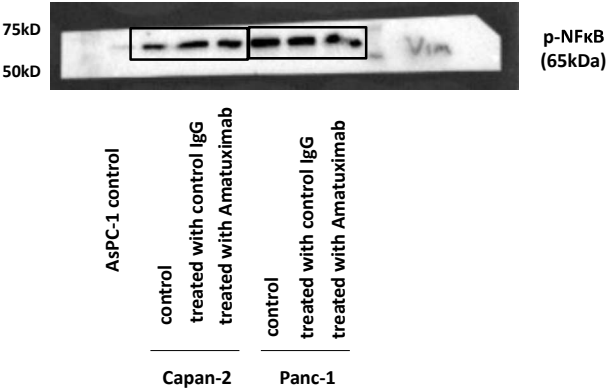

NKκB

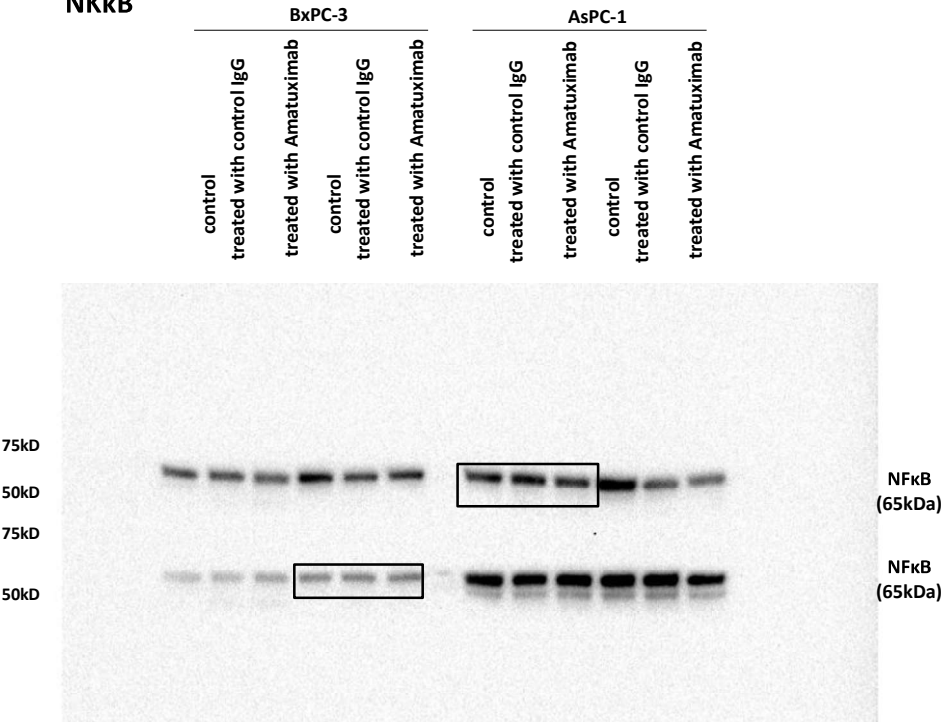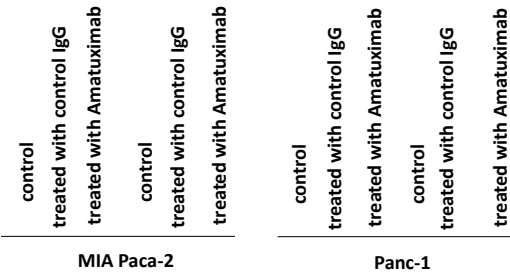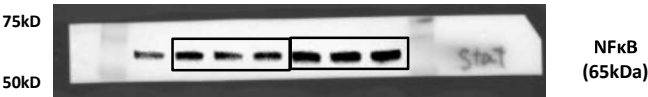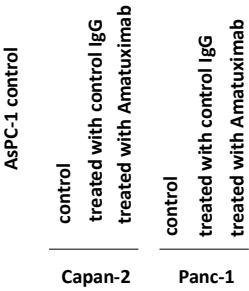

p-Stat3

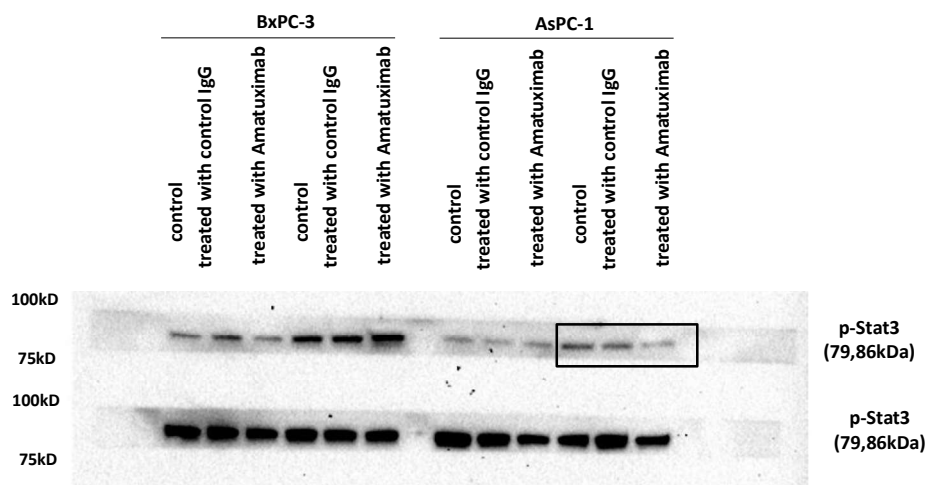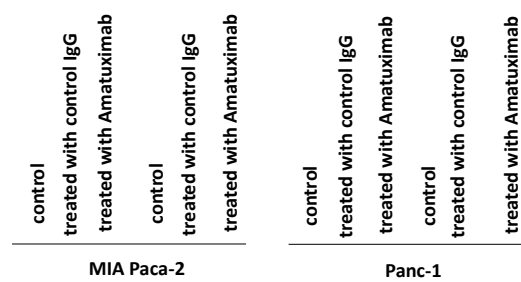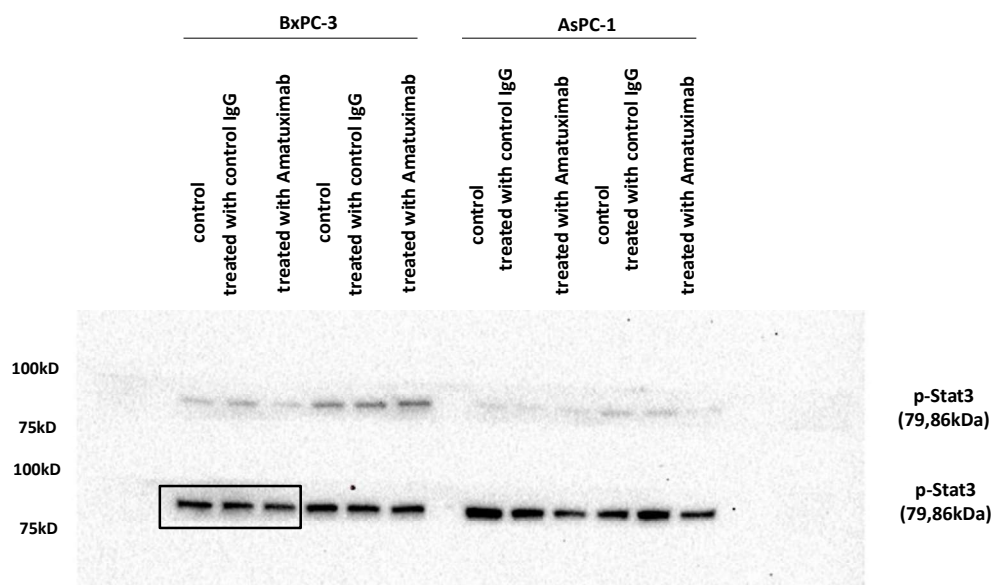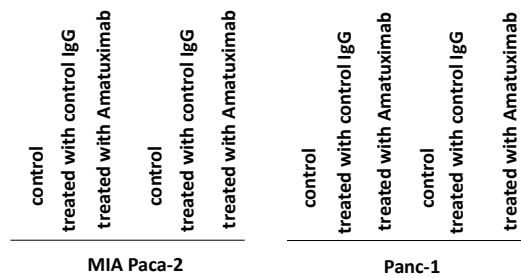

pStat3

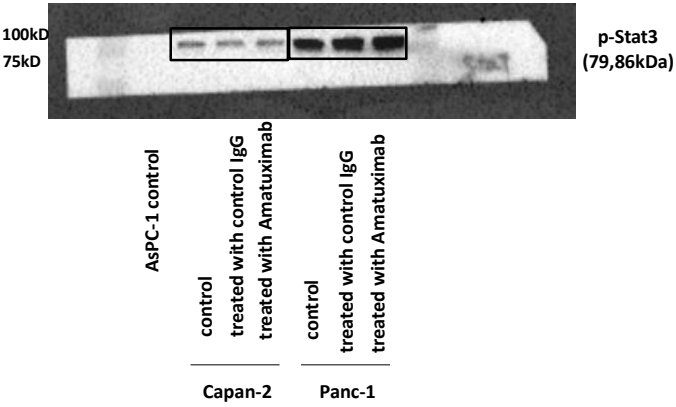

Stat3

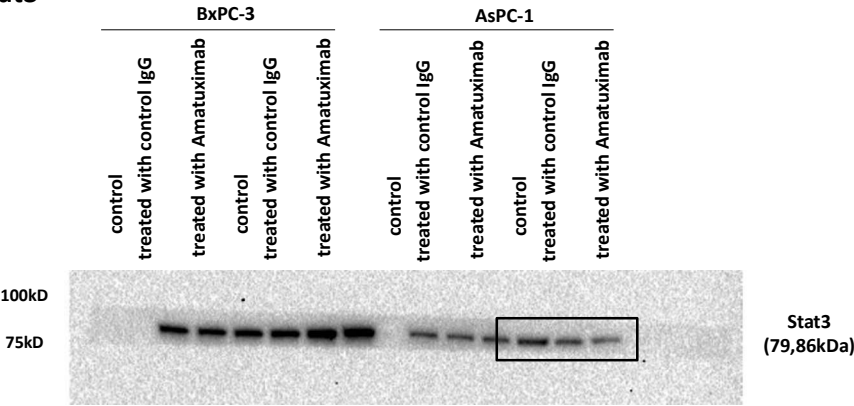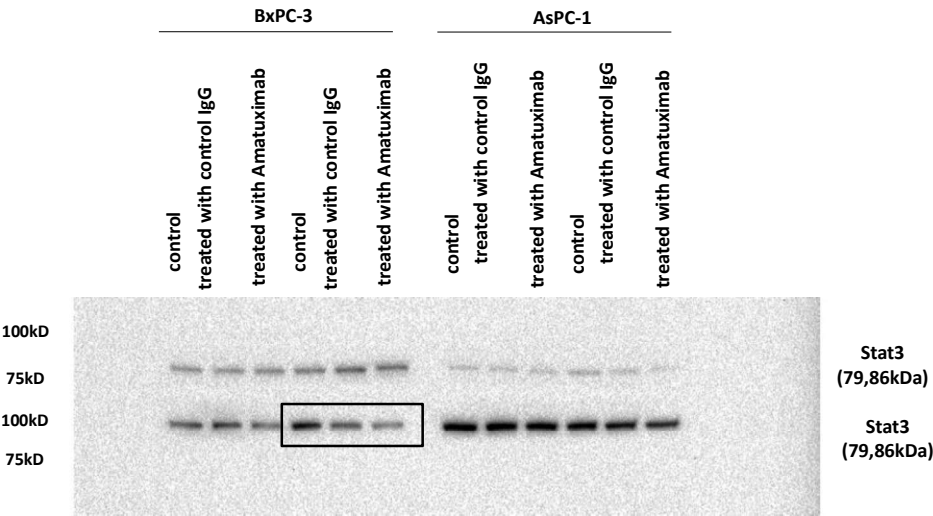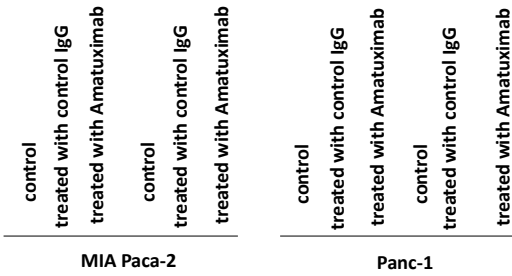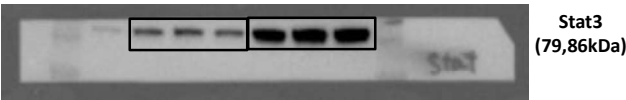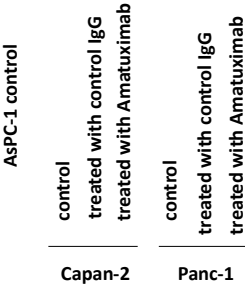

Supplement: Supplementary file 6 — Additional file 6: Supplemental Figure 4. Corresponding uncropped full-length blot images for Fig. 3b. The cropped blots were marked with black frame. Densitometric analysis of western blots was performed using a ChemiDoc XRS Plus system with Image Lab Software (Bio-Rad, Hercules, CA, USA). We cut the membranes according to the standard protein size markers and detected the blot using the images in those the blotting picture and marker were merged. [file 12885_2020_7722_MOESM6_ESM.pdf]

Supplemental figure 5

Figure 3C uncropped images

mesothelin

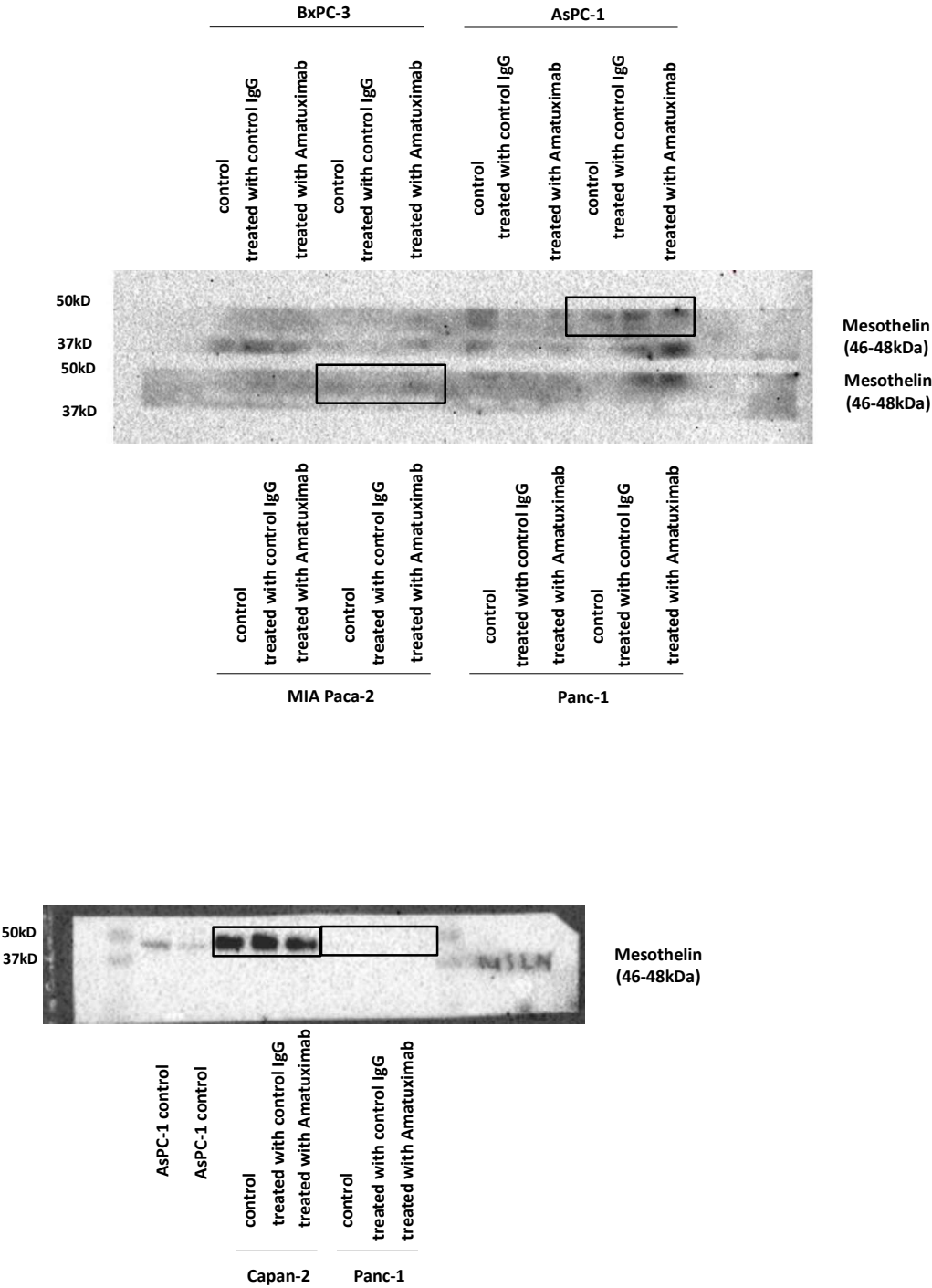

E-Cadherin

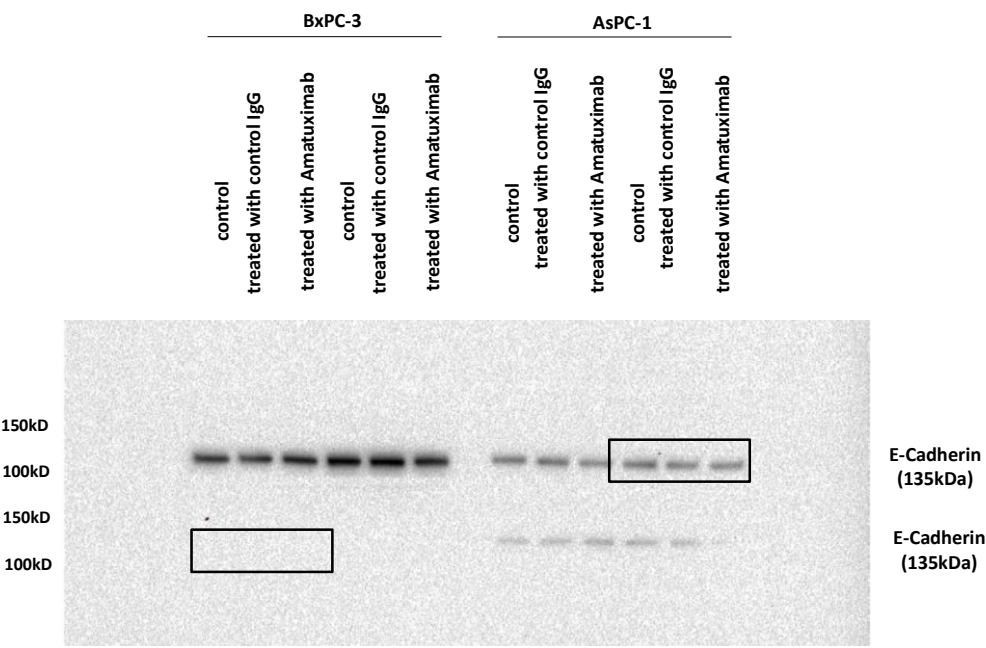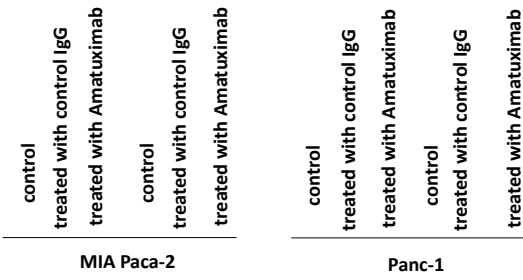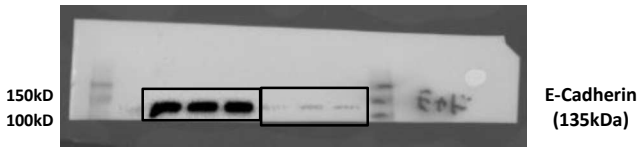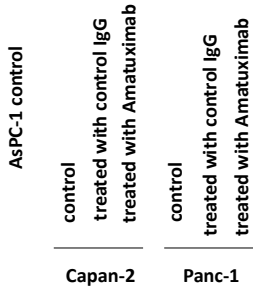

N-Cadherin

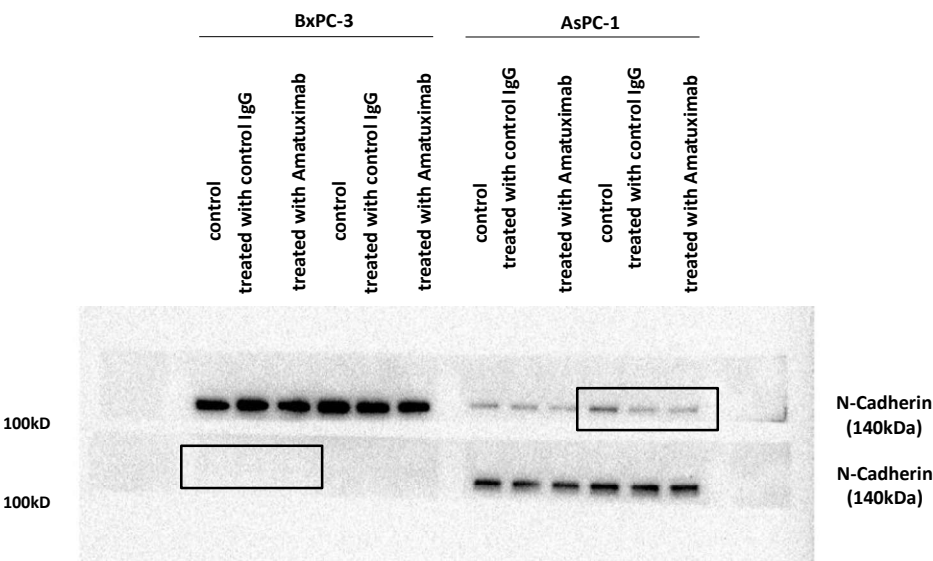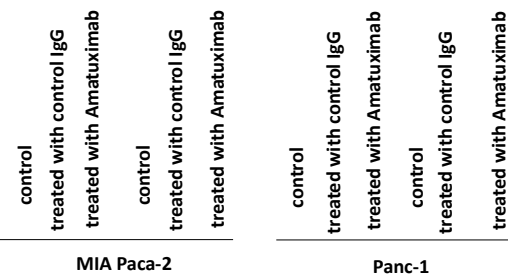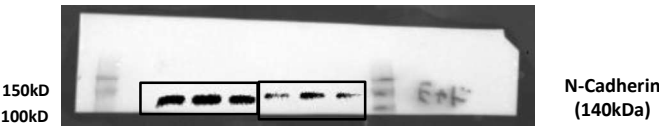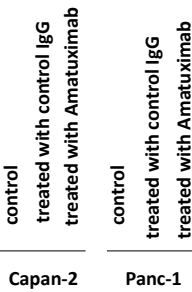

Vimentin

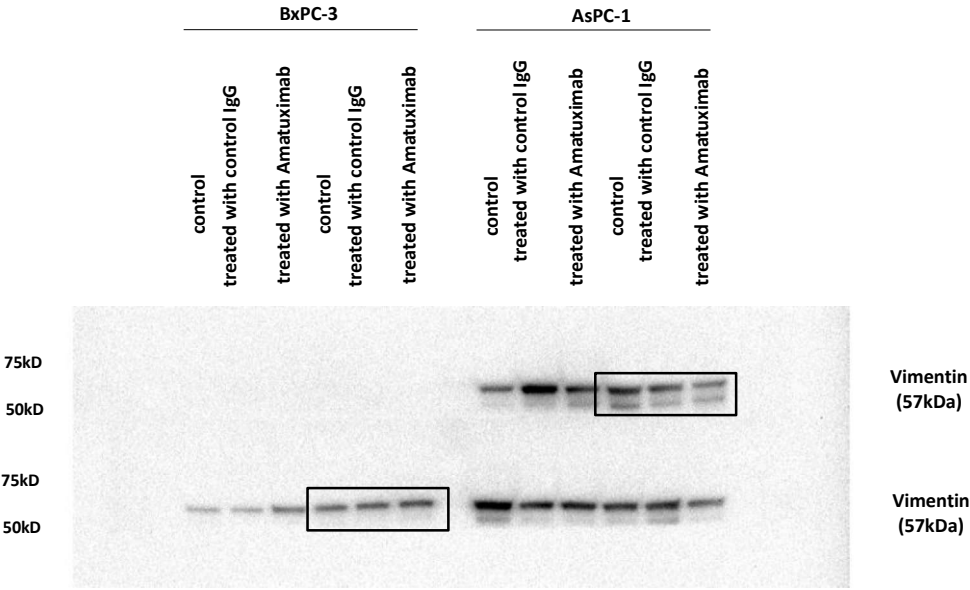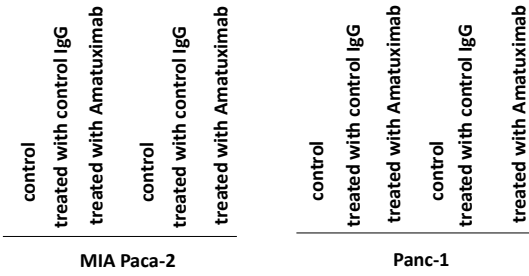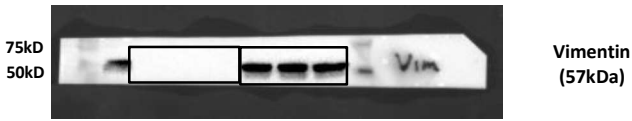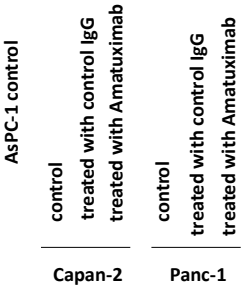

ZO-1

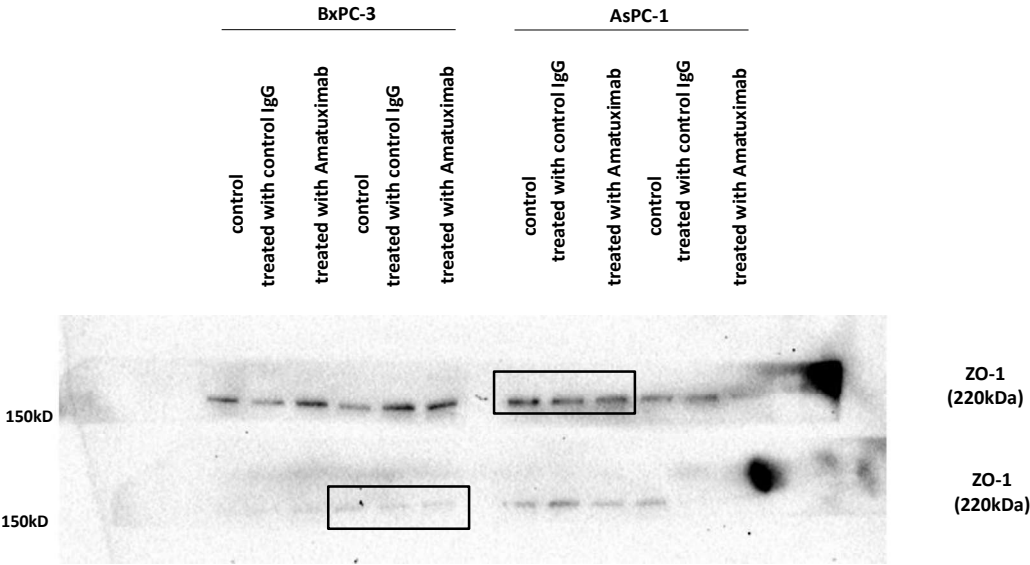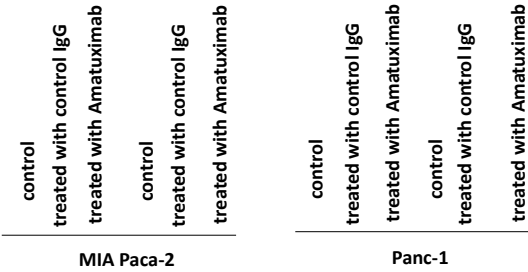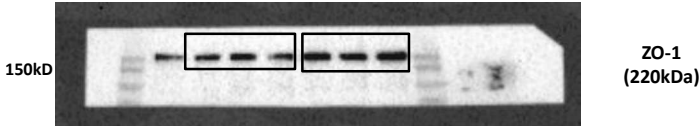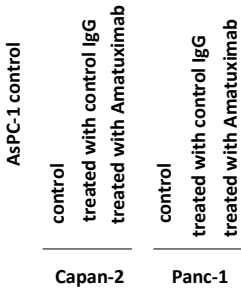

Supplement: Supplementary file 7 — Additional file 7: Supplemental Figure 5. Corresponding uncropped full-length blot images for Fig. 3c. The cropped blots were marked with black frame. Densitometric analysis of western blots was performed using a ChemiDoc XRS Plus system with Image Lab Software (Bio-Rad, Hercules, CA, USA). We cut the membranes according to the standard protein size markers and detected the blot using the images in those the blotting picture and marker were merged. [file 12885_2020_7722_MOESM7_ESM.pdf]

Supplemental figure 1 uncropped images

Mesothelin

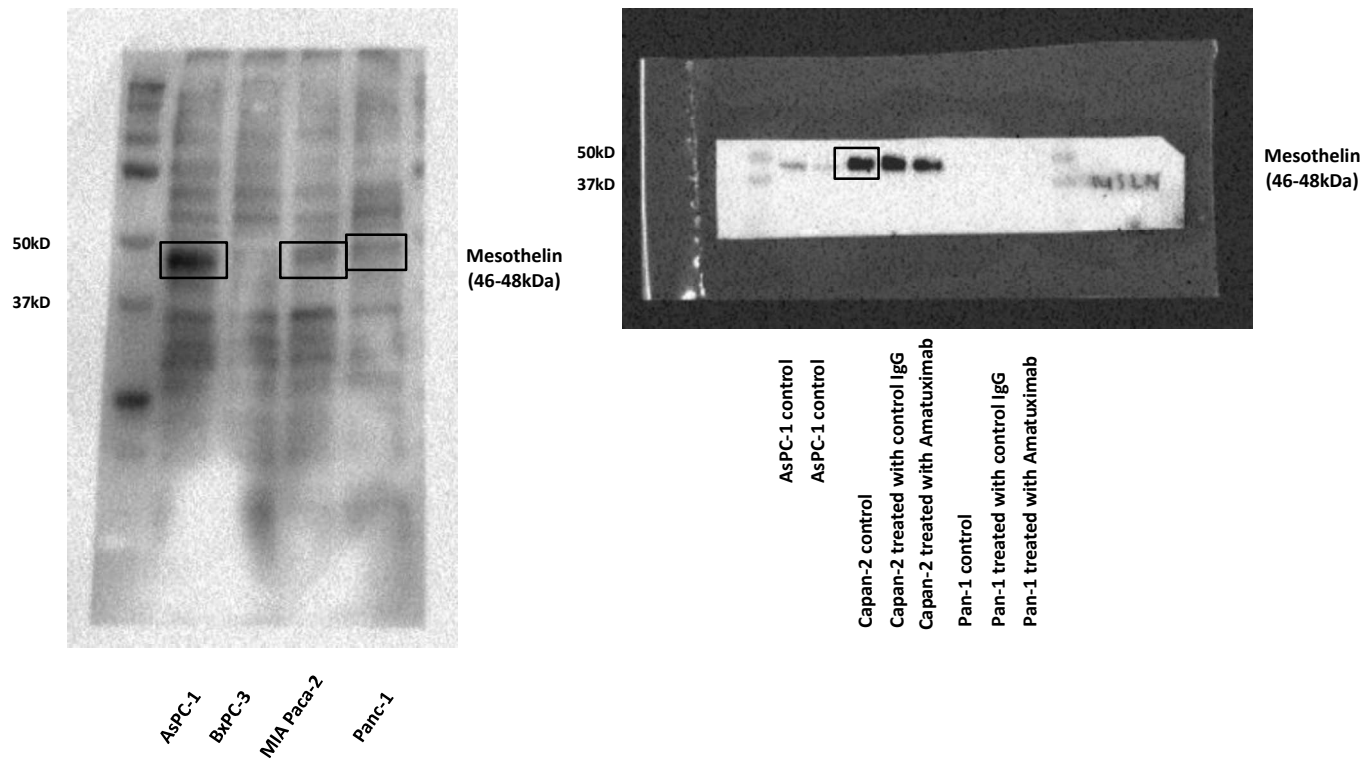

GAPDH

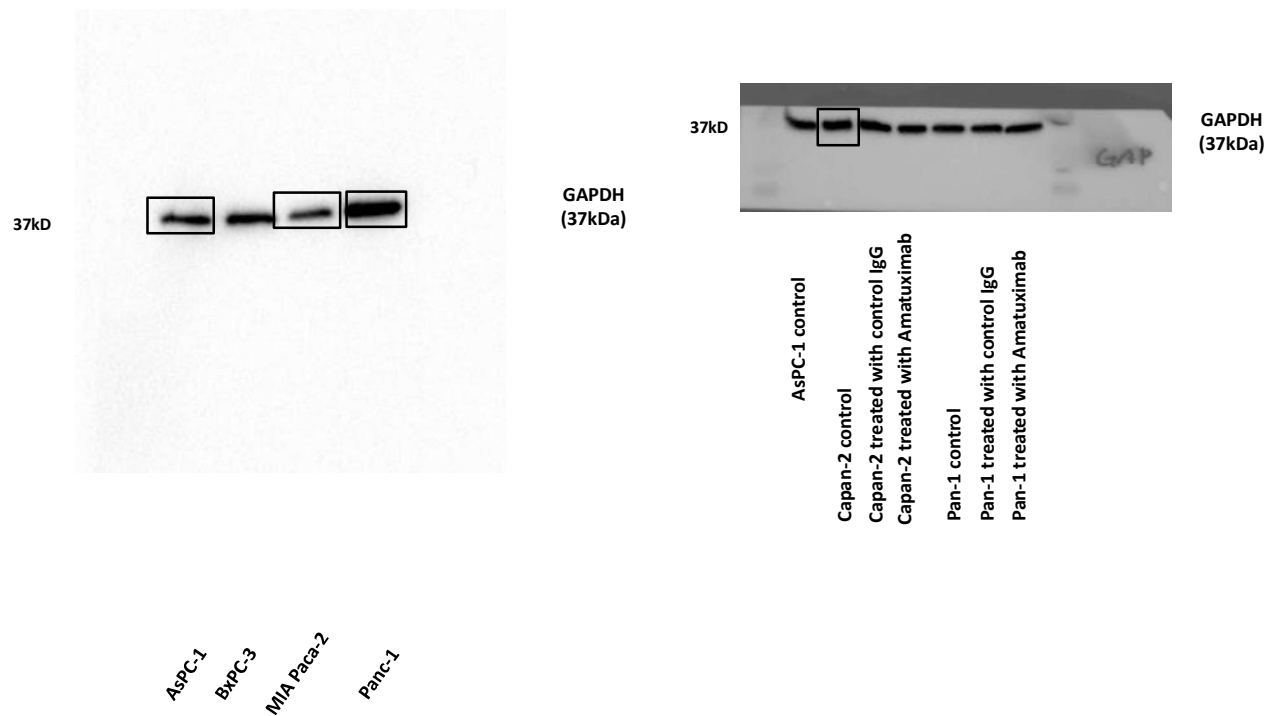

Supplement: Supplementary file 8 — Additional file 8: Supplemental Figure 6. Corresponding uncropped full-length blot images for supplemental Figure 1. The cropped blots were marked with black frame. Densitometric analysis of western blots was performed using a ChemiDoc XRS Plus system with Image Lab Software (Bio-Rad, Hercules, CA, USA). We cut the membranes according to the standard protein size markers and detected the blot using the images in those the blotting picture and marker were merged. [file 12885_2020_7722_MOESM8_ESM.pdf]
