# Supplementary material for: Mesothelin blockage by Amatuximab suppresses cell invasiveness, enhances gemcitabine sensitivity and regulates cancer cell stemness in mesothelin-positive pancreatic cancer cells
Source: BMC Cancer. 2021 Feb 26;21:200. doi: 10.1186/s12885-020-07722-3 (PMC7912898; doi:10.1186/s12885-020-07722-3)

Supplemental figure 7

Analysis of mesothelin expression in the four human pancreatic cancer cells by immunocytochemistry

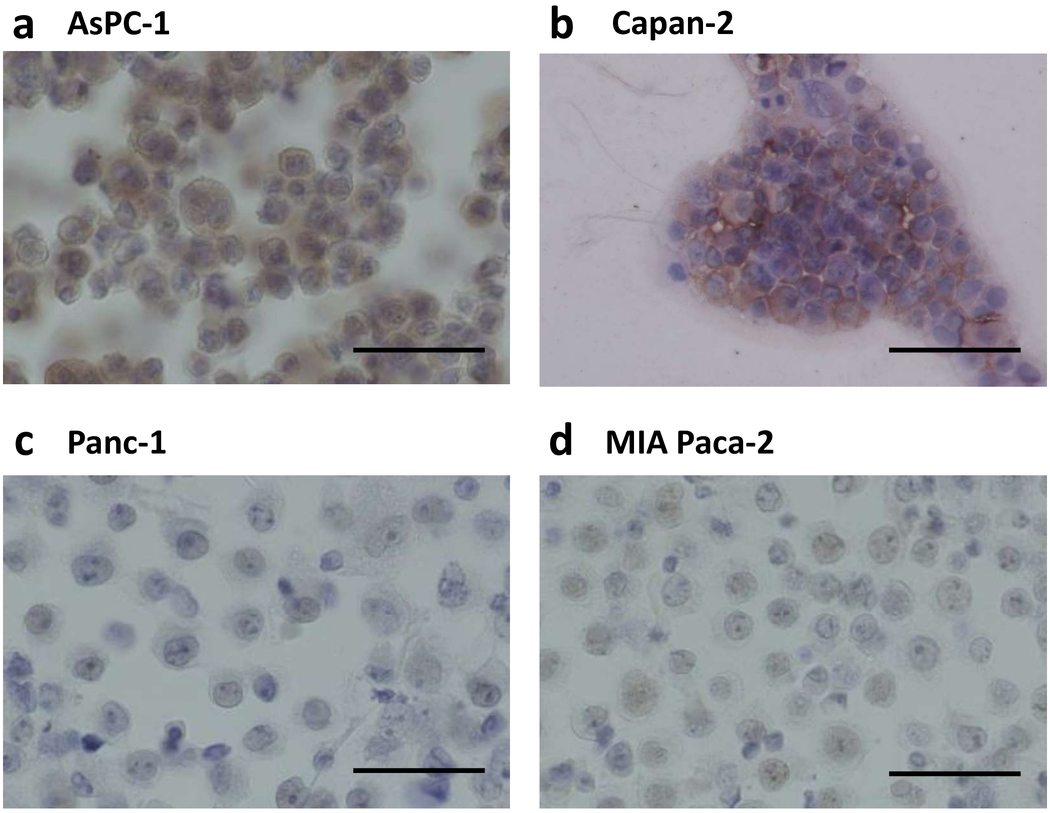

Supplement: Supplementary file 9 — Additional file 9: Supplemental Figure 7. Analysis of mesothelin expression in the four human pancreatic cancer cells by immunocytochemistry: (a) AsPC-1, (b) Capan-2, (c) Panc-1 and (d) MIA Paca-2 cells. The image of Capan-2 was taken by deferent researcher in another time, so in a little bit deferent condition. Scale bar, 100 μm. [file 12885_2020_7722_MOESM9_ESM.pdf]
